# Inhibition of SF3B1 improves the immune microenvironment through pyroptosis and synergizes with αPDL1 in ovarian cancer

**DOI:** 10.1038/s41419-023-06301-1

**Published:** 2023-11-27

**Authors:** Shourong Wang, Yao Liu, Huimin Xiao, Zhongshao Chen, Xiaohang Yang, Jingjing Yin, Yingwei Li, Cunzhong Yuan, Shi Yan, Gang Chen, Qinglei Gao, Beihua Kong, Chaoyang Sun, Kun Song

**Affiliations:** 1 Department of Obstetrics and Gynecology, Qilu Hospital of Shandong University, Jinan, 250012 China; 2 Gynecologic Oncology Key Laboratory of Shandong Province, Qilu Hospital of Shandong University, Jinan, 250012 China; 3 Department of Obstetrics and Gynecology, Tongji Hospital, Tongji Medical College, Huazhong University of Science and Technology, Wuhan, 430030 China

**Keywords:** Ovarian cancer, Immunotherapy, Cancer microenvironment

## Abstract

Ovarian cancer is resistant to immune checkpoint blockade (ICB) treatment. Combination of targeted therapy and immunotherapy is a promising strategy for ovarian cancer treatment benefit from an improved immune microenvironment. In this study, Clinical Proteomic Tumor Analysis Consortium (CPTAC) and The Cancer Genome Atlas (TCGA) cohorts were used to screen prognosis and cytotoxic lymphocyte infiltration-associated genes in upregulated genes of ovarian cancer, tissue microarrays were built for further verification. In vitro experiments and mouse (C57/BL6) ovarian tumor (ID8) models were built to evaluate the synergistic effect of the combination of SF3B1 inhibitor and PD-L1 antibody in the treatment of ovarian cancer. The results show that SF3B1 is shown to be overexpressed and related to low cytotoxic immune cell infiltration in ovarian cancer. Inhibition of SF3B1 induces pyroptosis in ovarian cancer cells and releases mitochondrial DNA (mtDNA), which is englobed by macrophages and subsequently activates them (polarization to M1). Moreover, pladienolide B increases cytotoxic immune cell infiltration in the ID8 mouse model as a SF3B1 inhibitor and increases the expression of PD-L1 which can enhance the antitumor effect of αPDL1 in ovarian cancer. The data suggests that inhibition of SF3B1 improves the immune microenvironment of ovarian cancer and synergizes ICB immunotherapy, which provides preclinical evidence for the combination of SF3B1 inhibitor and ICB to ovarian cancer treatment.

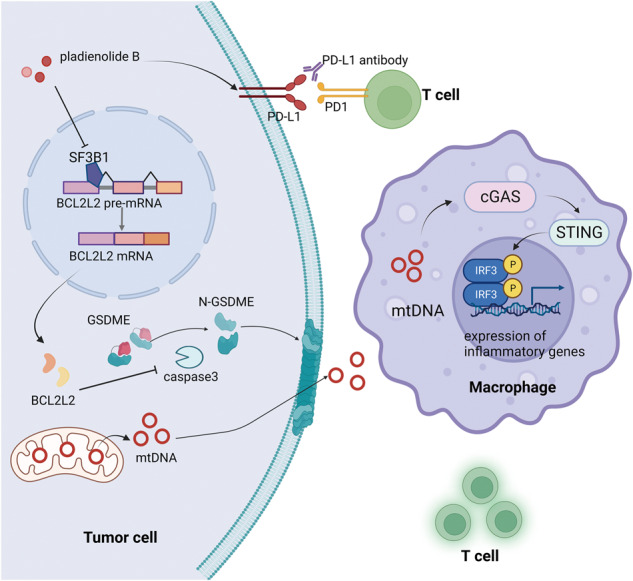

## Introduction

Advanced ovarian cancer has a poor prognosis and a lack of effective treatment, making it the fifth leading cause of cancer-related deaths of women in the United States [1]. Immune checkpoint blockade (ICB) monotherapy is less effective in ovarian cancer than in other solid tumors [2, 3]. Different responses to ICB may be attributed to several factors that directly or indirectly affect cytotoxic T lymphocyte (CTL) recruitment and activation, including the tumor mutational burden, expression of programmed cell death protein 1 (PD-1) and programmed death ligand 1 (PD-L1), homologous repair deficient and proficient phenotypes, and the tumor microenvironment (Tregs, M2-type tumor-associated macrophages (TAMs), plasmacytoid DCs, N2-type neutrophils, and myeloid-derived suppressor cells (MDSCs)) [4, 5]. Studies on the combination of ICB therapy with traditional chemotherapy, radiotherapy, and targeted treatment didn’t present inspiring achievement: A phase 2 clinical trial of a combination of nivolumab and bevacizumab in relapsed ovarian cancer demonstrated an objective response rate (ORR) of 28.9% and median progression-free survival (PFS) of 8.1 months [6]; JAVELIN Ovarian 100, a phase III randomized controlled trial (RCT) of chemotherapy-naïve epithelial ovarian cancer comparing chemotherapy alone versus chemotherapy followed by avelumab maintenance versus chemotherapy plus avelumab maintenance, was terminated early at a planned interim analysis for failure to meet its primary endpoint [7]. New immunotherapy combination regimens for ovarian cancer remain to be urgently developed.

Splicing Factor 3b Subunit 1 (SF3B1) is the largest subunit of the splicing factor 3b (SF3b) protein complex and is a constituent of U2 small nuclear ribonucleoprotein (U2snRNP) [8]. Mutations in SF3B1 are the most frequent and relevant spliceosome mutations in hematological diseases, including myelodysplastic syndromes [9]. SF3B1 mutations cause aberrant splicing and activation of inflammatory immune signaling in myelodysplastic syndromes [10–12]. SF3B1 promotes malignancy in pancreatic cancer, endometrial cancer, and glioblastoma [13–15]. Pladienolide B is an SF3B1 inhibitor widely used in cancer research. Pharmacological inhibition of SF3B1 by pladienolide B reduces tumor growth in cervical cancer, hepatocellular cancer, glioblastoma, chronic lymphocytic leukemia, and endometrial cancer [14–18].

Pyroptosis is a gasdermin-mediated programmed necrotic cell death, characterized by the release of inflammatory molecules and immune activation [19]. The induction of pyroptosis may be a promising strategy for antitumor immunity. Wang et al. found that pyroptosis in less than 15% of tumor cells was sufficient to clear the entire 4T1 mammary tumor graft [20]. Pyroptosis increases the infiltration of CD4 +, CD8 +, natural killer (NK), and M1 macrophage cell populations and decreases the populations of monocytes, neutrophils, MDSCs, and M2 macrophages in mammary tumors. Furthermore, the expression of gasdermin E (GSDME) can enhance tumor cell phagocytosis by TAMs and increase the number and function of tumor-infiltrating NK cells and CD8 + T lymphocytes [20, 21].

Here, we explored the relationships among SF3B1, pyroptosis, and cytotoxic immune cell infiltration in ovarian cancer. BCL2-like 2 (BCL2L2) was found as a key link regulated through splicing regulation by SF3B1, and mitochondrial DNA (mtDNA) released from tumor cells with pyroptosis played a crucial part in polarization of macrophages and infiltration of cytotoxic lymphocytes. Furthermore, combination of SF3B1 inhibitor and αPDL1 showed excellent efficacy in mice ovarian cancer models, indicating a good clinical application prospect in immunotherapy in ovarian cancers.

## Methods

### Cell lines and cell culture

A2780 and OVCAR8 cells were obtained from the M.D. Anderson Cancer Center characterized Cell line Core. ID8 cells, a murine ovarian cancer cell line derived from spontaneous malignant transformation of C57BL/6 mouse (RRID:MGI:2159769) ovarian surface epithelium cells was a gift from K. Roby (University of Kansas, Lawrence, KS). A2780 and OVCAR8 cells were cultured with RPMI-1640 medium plus 10% FBS. ID8 cells were cultured with DMEM medium plus 10% FBS. ID8^△mtDNA^ cells were established by ID8 cells with 50 ng/mL ethidium bromide, 100 μg/mL sodium pyruvate, and 50 μg/mL uridine treatment for 3 weeks. ID8^△mtDNA^ cells were cultured in DMEM medium with 100 μg/mL sodium pyruvate, 50 μg/mL uridine, and 10% FBS. All cells were maintained in humidified incubators under 37 °C, 5% CO_2_. Moreover, cell lines were regularly tested for mycoplasma before any experiments were performed, and the identities of the cell lines were confirmed by short tandem repeat (STR) analysis.

### Antibodies and compounds

Antibodies used in the study are listed in Table S1.

Pladienolide B (sc-391691) was purchased from Santa Cruz and dissolved in DMSO. anti-PD-L1 antibody (BE0101, clone B7-H1) and IgG isotype control (BE0090) were from BioXcell. CD45 (APC-cy7, 103115), CD3 (PerCP/Cyanine5.5, 100217), CD4 (FITC, 100405), CD25 (PE, 102007), FOXP3 (Alexa Fluor® 647, 320013), CD8 (FITC, 100706), IFN-γ (PE, 505807), CD11b (PE/Cyanine7, 101215), F4/80 (FITC, 123107), CD163 (PE, 156703), CD86 (PerCP/Cyanine5.5, 105027), Ly-6G (APC, 127613) were from BioLegend. Clodronate Liposomes were from Yeasen.

### Immunohistochemical staining (IHC)

Tissue microarray samples were sourced from 261 patients at Qilu Hospital of Shandong University with comprehensive clinical records, encompassing fallopian tube and high-grade serous ovarian cancer tissues. Informed consent was obtained from all participants. IHC staining of tissue microarray (TMA) and tumor tissue were carried out according to the manufacturer’s instructions. In brief, tissues were deparaffinized, antigen-retrieved, and stained overnight at 4 °C with primary antibodies. Primary antibodies included SF3B1 (1:200), CD8 (1:1000), and PD-L1 (1:100). Following secondary antibody incubation and diaminobenzidine staining, the slides were examined with a microscope and photographs taken. We scored staining intensity as 0 (negative), 1 (weak), 2 (moderate), and 3 (strong). The extent of staining was determined by the percentage of positive tumor cells: 1 (0–25%), 2 (26–50%), 3 (51–75%), and 4 (76–100%). IHC staining intensity and percentage scores were multiplied to generate the final score. Two experts, blinded to the clinical and prognostic information of the patients, independently determined the immunohistochemical staining scores under identical conditions. In the tissue microarray samples, tissues that had fallen off or had incomplete prognostic information were excluded from the analysis. In rare cases, inconsistent scores were reassessed by another expert.

### ID8 mouse tumor model

All animal experiments had been approved by the Animal Experiment Ethics Committee of Qilu Hospital. We estimated the required mouse sample size using the resource equation method, ultimately determining a total of 12 mice (divided into 2 groups) and 24 mice (divided into 4 groups) for our experiments. C57BL/6 mice (female, 6–8 weeks old) were purchased from Beijing Charles River Biotechnology and were used for ID8 tumor model. 2 × 10^5^ ID8 cells were subcutaneously injected into right flank of the C57BL/6 mice in a 1:1 mix of PBS and Matrigel. On the third day after injection, the mice were randomly grouped and given the corresponding treatments. In Fig. 2a, treatment cohorts were randomized as follows: vehicle (10% DMSO, every 3 days for six times) and pladienolide B (0.5 mg/kg, every 3 days for six times) (*n* = 6 each group). When the tumors were palpable (about 50 mm^3^), tumor volumes were evaluated every 3 days. In Fig. 5a, treatment cohorts were randomized as follows: vehicle (10% DMSO and PBS control), pladienolide B (0.5 mg/kg, every 3 days for six times), Clodronate Liposomes (150 μL/mouse every 2 weeks) and doublet (*n* = 6 each group). In Fig. 7c, treatment cohorts were randomized as follows: vehicle (10% DMSO and IgG isotype control), αPDL1(200 µg/mouse, every 3 days, 6 times), pladienolide B (0.5 mg/kg, every 3 days, 6 times) and doublet (*n* = 6 each group). The animal studies were executed utilizing a single-blind experimental design.

### Flow cytometry and cell markers staining

Tumors were washed by PBS and were cut into small pieces and then incubated in enzymes to dissociate into single cells followed the instructions of the Tumor Dissociation Kit (Miltenyi Mouse Tumor Dissociation Kit (RRID:SCR_020285)). Tumor tissue samples were equally divided into three parts, one part was used for staining CD45, CD3, CD4, CD25, FOXP3, another was used for staining CD45, CD3, CD8, IFN-γ, and the third was used for staining CD45, CD11b, F4/80, CD163, CD86, Ly6G. Staining was performed with 1 μL/sample fluorochrome-labeled antibodies of surface markers in staining buffer at 4 °C for 30 min. Next, the cells were fixed, permeabilized, and stained with anti-Foxp3 and anti-IFN-γ antibodies. CytoFlex flow cytometry was used to analyze single-cell suspensions of tumor tissue. The information of antibodies was listed as Table S1 in supplementary materials.

### dsDNA and dsRNA staining

Picogreen dsDNA Quantitation Reagent (Yeasen, cat#12641ES01) was used to stain dsDNA, in brief, picogreen were 1:200 diluted with culture medium and co-incubation with cells for 2 h. Then cells were washed by PBS and fixed with DAPI counterstaining and observed under fluorescence microscope.

DMSO or pladienolide B-treated cells were fixed and permeabilized. After blocking with BSA, cells were incubated with J2 antibody (Biocompare; cat #10010500) at dilution of 1:200 at 4 °C overnight. Fluorescent secondary antibody and DAPI staining were performed before taking pictures under a microscope.

### Colony formation assay

ID8, OVCAR8, and A2780 cells were seeded into six-well plates (400 cells per well) and treated with corresponding concentrations of pladienolide B. After 2 weeks of incubation, cells were fixed with methanol and stained with 0.1% crystal violet. Quantification was achieved by counting the number of cell colonies. The data were presented as the mean ± SD and represented three independent experiments.

### Cell viability analysis

Cell viability analysis was performed by CCK-8 Cell Counting Kit (vazyme, cat#A311-01). 3000–5000 cells were seeded into 96-well plates per well and treated with pladienolide B for 72 h. Then the cells were incubated with 100 μL fresh culture medium with 10% CCK8 solution in 37 °C for 3 h. Fresh media were used as a control. The absorbance at 450 nm was quantified by a microplate spectrophotometer (BioRad).

### Western blot

Cells were collected, added RIPA lysis buffer and ultrasonically lysed to extract protein, the protein concentration was determined by BCA method. A total of 20 μg protein was added to each well and performed SDS-PAGE electrophoresis. Proteins were transferred to PVDF membranes and blocked with 5% nonfat milk. The membranes were incubated in primary antibodies at 4 °C overnight followed by 1:5000 incubation of HRP-labeled secondary antibody. The ECL system (GE Healthcare) was used to detect the expression of protein. The information of antibodies was listed as Table S1 in supplementary materials.

### LDH release analysis

The release of LDH was analyzed by LDH Release Assay Kit (Beyotime Biotechnology, cat#C0016) according to manufacture introduction. 3000–5000 cells were seeded into 96-well plates per well and incubated with pladienolide B for 72 h. One hour prior to detection, cell lysis buffer was added to the positive control well to release LDH. Cell culture plate was centrifuged at 400 × *g* for 5 min and the cell culture supernatant was transferred to a new 96-well plate. Fresh medium was blank control. The newly prepared detection solution was added to the 96-well plate and incubated for 30 minutes at dark. The absorbance at 490 nm was quantified by a microplate spectrophotometer (BioRad). The release of LDH was calculated by (absorbance of sample − absorbance of blank)/(absorbance of positive control-absorbance of blank) × 100%.

### ELISA assay

ELISA assay kit of IL18 and IL1β were purchased from ABclonal (cat#RK00176, #RK00001). Culture supernatants of A2780 and OVCAR8 treated with different concentration of pladienolide B were collected and added to detection wells. Freshly prepared standard solutions were also added to detection wells. The detection plate was incubated at 37 °C for 90 min. After washing, biotin-labeled secondary antibody was added and the plated was incubated at 37 °C for 60 min away from light. After working solution, substrate solution and stop solution added to detection wells in sequence according to manufacture introduction, The absorbance at 450 nm was quantified by a microplate spectrophotometer (BioRad).

### RIP-PCR

RIP assay was performed using Magna RIP RNA-Binding Protein Immunoprecipitation Kit (cat#17-700) purchased from Milipore. OVCAR8 cells treated with DMSO or pladienolide B were collected to perform RIP assay. Cells were lysed and incubated with SF3B1 antibody (LifeSpan, 1:100) and magnetic beads. RNA was extracted from immunocomplexes and detected by qPCR. Primer sequences were listed as Table S2 in supplementary materials.

### qPCR

RNA was extracted using TRIzol regent according to the manufacturer’s protocol. RNA was reverse transcribed into cDNA using the HiScript II Q RT SuperMix for qPCR Kit (Vazyme, cat#R233-01). qPCR was performed using SYBR qPCR Master Mix (Vazyme, cat#Q711-02). Relative gene expression was determined by ΔΔCt method and normalized by GAPDH. Primer sequence was listed as Table S3 in supplementary materials.

### RNA-pulldown assay

RNA-pulldown assay was performed using the RNA pull-down kit (GENESEED, cat#P0201) according to the manufacturer’s protocol. In brief, BCL2L2 fragments were amplified by PCR and biotin-labeled by RNAmax-T7 Biotin Labeled Transcription Kit (RiboBio, cat#C11002-1). Biotin-labeled RNA was incubated with streptomycin magnetic beads and OVCAR8 cell lysate. Protein was extracted and detected by immunoblotting.

### Cytoxicity of NK cell, T cell, and BMDM

NK cells were separated by MojoSort Mouse NK Cell Isolation Kit (Biolegend, cat#480049) from mouse spleen cells. T cells were separated by MojoSort Mouse CD3 T Cell Isolation Kit (Biolegend, cat#480023) from mouse spleen cells. BMDM were separated from mouse bone marrow cells by incubation with 10 ng/mL M-CSF for 5–7 days.

Cytoxicity of T cell and BMDM was measured by ID8-Luc cells and cytoxicity of NK cells was measured by YAC-1-Luc cells. T cells or BMDM were co-cultured with ID8-Luc cells at ratio of 20:1, 10:1, 5:1, and 2.5:1. NK cells were co-cultured with YAC-1-Luc at ratio of 20:1, 10:1, 5:1, and 2.5:1. DMSO or pladienolide B or supernatant of ID8 treated with DMSO or pladienolide B was added into co-culture system. After 12–24 h incubation, luciferase activity was measured by Bright-Lumi II Firefly Luciferase Assay Kit (Beyotime, cat#RG052S). ID8-Luc alone or YAC-1-Luc alone was used as control.

### mPTP openness assay

mPTP openness was measured by mPTP Assay Kit (Beyotime, cat#C2009S). A2780, OVCAR8, and ID8 cells treated with DMSO or pladienolide B were collected and detected by flow cytometry according to manufacturer’s protocol.

### DNA extraction from supernatant

DNA was extracted from supernatant of OVCAR8 and ID8 cells treated with DMSO or pladienolide B by FastPure Blood/Cell/Tissue/Bacteria DNA Isolation Mini Kit (Vazyme, cat#DC112-01) according to manufacturer’s protocol.

### mtDNA extraction and labeling

Mitochondria of ID8 cells was separated by Cell Mitochondria Isolation Kit (Beyotime, cat#C3601) according to manufacturer’s protocol. DNA in mitochondria was extracted using FastPure Blood/Cell/Tissue/Bacteria DNA Isolation Mini Kit (Vazyme, cat#DC112-01). mtDNA was labeled by cy3-dCTP (Cytiva, cat#PA53021) using Biotin Random Prime DNA Labeling Kit (Beyotime, cat#D3118) according to manufacturer’s protocol. Labeled mtDNA was incubated with BMDM of mouse. BMDM was stained by F4/80 antibody (ABclonal, cat#A18637, 1:100) and the phagocytosis of mtDNA by BMDM was observed under fluorescence microscope.

### RNA-Seq analysis

OVCAR8 cells treated with DMSO or pladienolide B for 72 h were collected and performed next-generation sequencing by the Ribobio Biotechnology Company. The RNA-seq data generated in this study have been deposited in the NCBI GEO database under the accession number GSE222199. Raw reads were trimmed, and clean data was aligned to hg38 using hisat2 [22]. Reads per kilobase of exon model per million mapped reads (RPKM) normalized bigwigs were generated by deepTools [23]. DESeq2 was used to analyze differential expression genes between groups [24]. GSEA was performed with R package ClusterProfiler [25].

### Statistical analysis

The differences between two groups were calculated using the unpaired two-tailed Student’s t test (data followed a normal distribution). The log-rank test was used to detect differences in clinical prognosis. One-way ANOVA with Bonferroni post hoc test was used to compare differences between multiple groups. We employed both the F-test and the Brown-Forsythe test to assess the homogeneity of variances across the groups. Pearson’s correlation coefficient was used to analyze correlations between variables, using a t test to assess the significance of the correlation. The threshold for statistical significance was *p* < 0.05. All statistical analyses were done using SPSS 17.0. Data were analyzed and plotted using GraphPad Prism 8 software. rMATS (4.1.2) was used to conduct alternative splicing analysis (A3SS, A5SS, MXE, RI, and SE, both Junction Counts and Exon Counts), on RNA-seq data with SF3B1 inhibition.

## Results

### SF3B1 is associated with poor prognosis and low cytotoxic immune cell infiltration in ovarian cancer

**Fig. 1:**
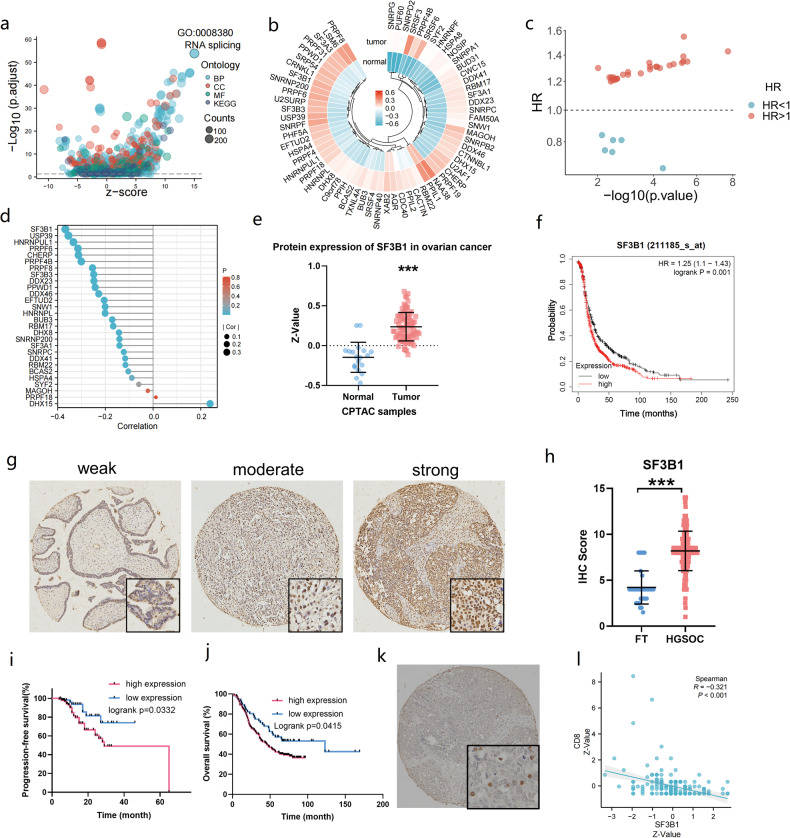
SF3B1 is associated with poor prognosis and lower cytotoxic immune cell infiltration in ovarian cancer. **a** Bubble diagram of GO and KEGG analysis of differentially expressed genes in normal and ovarian cancer of CPTAC cohort. **b** Circular heatmap of upregulated splicing factors in normal and ovarian cancer of CPTAC cohort. **c** Dot plot of the hazard ratio of upregulated splicing factors to progression-free survival in patients with SOC from the Kaplan–Meier Plotter database. **d** A lollipop chart of the correlation coefficient of cytotoxic immune cell infiltration with deleterious splicing factors (upregulated splicing factors with hazard ratios greater than one) in patients with SOC from the TCGA cohort. Cytotoxic immune cell infiltration score was calculated with sGSEA method. **e** SF3B1 protein level was analyzed in SOC (*n* = 84) and normal (*n* = 19) samples of CPTAC database. **f** Kaplan–Meier analysis of SF3B1 expression and progression-free survival of ovarian cancer patients from the Kaplan–Meier Plotter database. **g** Representative image of SF3B1 immunohistochemical staining on tissue microarray of ovarian cancer patients from Qilu Hospital. **h** Immunohistochemical score of normal fallopian tube (*n* = 35) and high-grade serous ovarian cancer (*n* = 226) from Qilu Hospital. **i**, **j** Correlation analysis of SF3B1 expression with progression-free survival (**i**) and overall survival (**J**) in ovarian cancer patients from Qilu Hospital. **k** Representative image of CD8 immunohistochemical staining on tissue microarray of ovarian cancer patients from Qilu Hospital. **l** Correlation analysis of SF3B1 and CD8 immunohistochemical score. *p* values were determined by non-parametric t tests. *p < 0.05. **p < 0.01. ***p < 0.001.

In order to screen targets that can improve the immune microenvironment of ovarian cancer, we analyzed differentially expressed proteins using the Pacific Northwest National Laboratory (PNNL) panel of the Clinical Proteomic Tumor Analysis Consortium (CPTAC) cohort. Gene ontology (GO) and Kyoto Encyclopedia of Genes and Genomes (KEGG) analyses showed that the RNA splicing pathway was the most enriched in ovarian cancer (Fig. 1a), and the upregulated splicing factors are shown in Fig. 1b. The hazard ratio of these upregulated splicing factors to PFS in ovarian cancer was calculated using the Kaplan–Meier plotter database with the best cutoff. As shown in Fig. 1c, among the 34 upregulated splicing factors with *p* values less than 0.01, six had hazard ratios less than 1, and 28 had hazard ratios greater than 1. The single-sample Gene Set Enrichment Analysis (ssGSEA) method was used to estimate immune cell infiltration. Then Pearson correlation coefficients between upregulated splicing factors with hazard ratios greater than 1 and cytotoxic immune cell infiltration score calculated by the ssGSEA method were analyzed in the TCGA cohort of ovarian cancer. As shown in Fig. 1d, SF3B1 expression exhibited the most pronounced negative correlation with cytotoxic immune cell infiltration. As shown in Fig. 1e, the protein level of SF3B1 was higher in ovarian cancer tissues (*n* = 19) than in normal tissues (*n* = 84) based on the PNNL panel of the CPTAC cohort. High levels of SF3B1 RNA expression were associated with poor PFS of ovarian cancer in the Kaplan–Meier Plotter database (Fig. 1f). To confirm the significance of SF3B1 in the prognosis of ovarian cancer, we performed immunohistochemistry using a tissue microarray (TMA) containing 35 normal fallopian tube samples and 226 high-grade serous ovarian cancer samples from Qilu Hospital (Jinan, China). As expected, the expression of SF3B1 was markedly increased in ovarian cancer (Fig. 1g, h), and high levels of SF3B1 were correlated with poor PFS (Fig. 1i) and overall survival (Fig. 1j). Moreover, infiltration of CD8 + T cells was also detected by immunohistochemistry using TMA (Fig. 1k) and the correlation of SF3B1 expression and CD8 + T cells infiltration was analyzed. As shown in Fig. 1l, SF3B1 expression was negatively correlated to CD8 + T cells infiltration. Taken together, the results indicated that SF3B1 is associated with poor prognosis and low cytotoxic immune cell infiltration in ovarian cancer.

### Inhibition of SF3B1 by pladienolide B prevents tumor growth and increases the proportion of cytotoxic lymphocyte infiltration in mouse allograft tumors

**Fig. 2:**
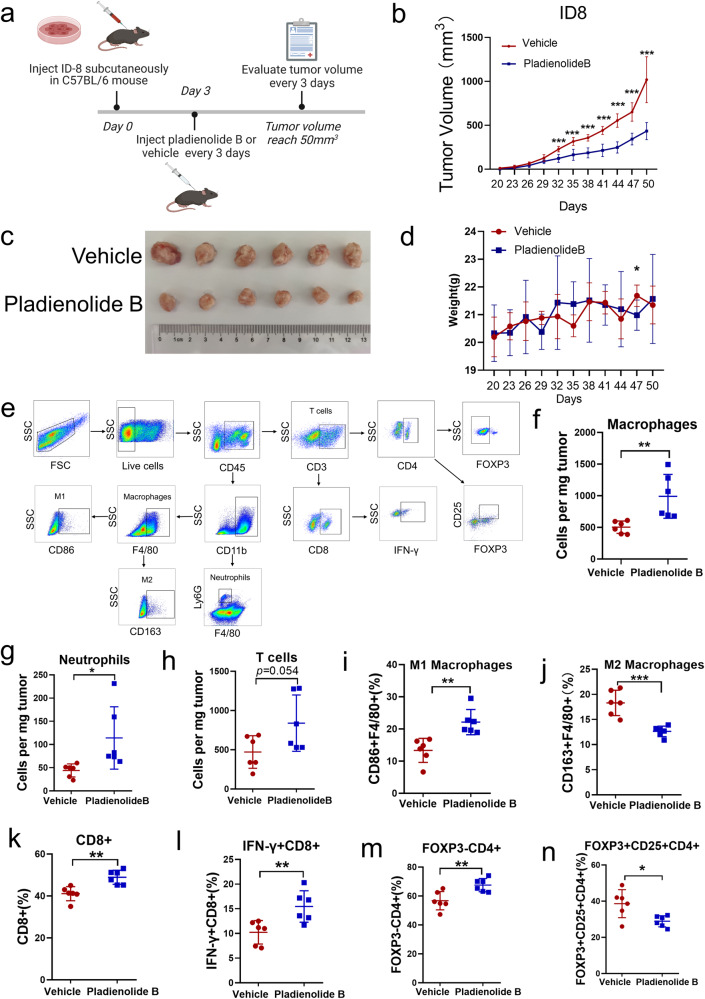
Targeted inhibition of SF3B1 by pladienolide B prevents tumor growth and increases cytotoxic lymphocyte infiltration. **a** Flow chart of mouse tumor inoculation and drug administration. ID8 cells (2 × 10^5^) were subcutaneously injected into the left groin of C56BL/6 mice (6–8 weeks old). After three days, mice were randomized into treatment cohorts accordingly (*n* = 6 for each group) and given drug treatment: vehicle (10% DMSO in PBS) or pladienolide B (0.5 mg/kg, every 3 days, 6 times). After the tumors were palpable, tumor volume was evaluated every 3 days. Treatment was stopped at day 18 and tumors growth was still monitored for another 5 weeks. **b** Growth curve of tumor volume. **c** Image of transplantation tumors in vehicle group and pladienolide B group. **d** Line chart of body weight of mice in vehicle group and pladienolide B group. **e** Diagram of flow cytometry gating strategies for tumor-infiltrating immune cells. **f** Amount of macrophage cells per milligram tumor in vehicle group and pladienolide B group. **g** Amount of macrophages per milligram tumor in vehicle group and pladienolide B group. **h** Amount of neutrophils per milligram tumor in vehicle group and pladienolide B group. **i** The proportion of CD86 + F4/80 + M1 macrophages to macrophages in vehicle group and pladienolide B group. **j** The proportion of CD163 + F4/80 + M2 macrophages to macrophages in vehicle group and pladienolide B group. **k** The proportion of CD8+ cells to CD3+ cells in vehicle group and pladienolide B group. **l** The proportion of IFN-γ + CD8+ cells to CD8+ cells in vehicle group and pladienolide B group. **m** The proportion of FOXP3-CD4+ cells to CD4+ cells in vehicle group and pladienolide B group. **n** The proportion of FOXP3 + CD25 + CD4+ Treg cells to CD4+ cells in vehicle group and pladienolide B group. *p* values were determined by non-parametric t tests. *p < 0.05. **p < 0.01. ***p < 0.001.

To further explore the effect of SF3B1 on immune cell infiltration of ovarian cancer, we injected the mouse ovarian cancer cells ID8 into C57BL/6 mice and administered pladienolide B, an SF3B1 inhibitor, or vehicle (Fig. 2a). Inhibition of SF3B1 by pladienolide B significantly prevented tumor growth (Fig. 2b, c), with no significant weight change in mice (Fig. 2d). Mouse tumor tissues were dissociated and tumor-infiltrating immune cells were stained and detected by flow cytometry (Fig. 2e). As illustrated in Fig. 2f, g, upon inhibition of SF3B1 using pladienolide B, there was a significant increase in the number of neutrophils and macrophages per mg of tumor tissue (*p* < 0.05). In addition, there was a modest increase in the number of T cells (*p* = 0.054) (Fig. 2h). As shown in Fig. 2k–m, the proportions of CD8 + T cells, IFN-γ + CD8 + T cells, and FOXP3-CD4+ cells increased after pladienolide B treatment, whereas the proportion of Treg cells (FOXP3 + CD25 + CD4 +) decreased (Fig. 2n). Moreover, following the inhibition of SF3B1, the proportion of M1 macrophages significantly increased, while the proportion of M2 macrophages notably decreased (*p* < 0.05) (refer to Fig. 2i, j). To further explore the mechanism of pladienolide B-induced antitumor immunity, we detected the aggregation of dsDNA and dsRNA in the cytoplasm after pladienolide B administration. As shown in Figure S1a, b, pladienolide B treatment did not increase the aggregation of dsDNA and dsRNA in the cytoplasm. Moreover, qPCR and GSEA analyses of RNA-seq results showed that pladienolide B failed to activate the interferon pathway in ovarian cancer (Figure S1c, d). Taken together, the results showed that the inhibition of SF3B1 by pladienolide B prevents tumor growth and induces antitumor immunity, but not through the interferon pathway.

### Inhibition of SF3B1 induces ovarian cancer cell pyroptosis

**Fig. 3:**
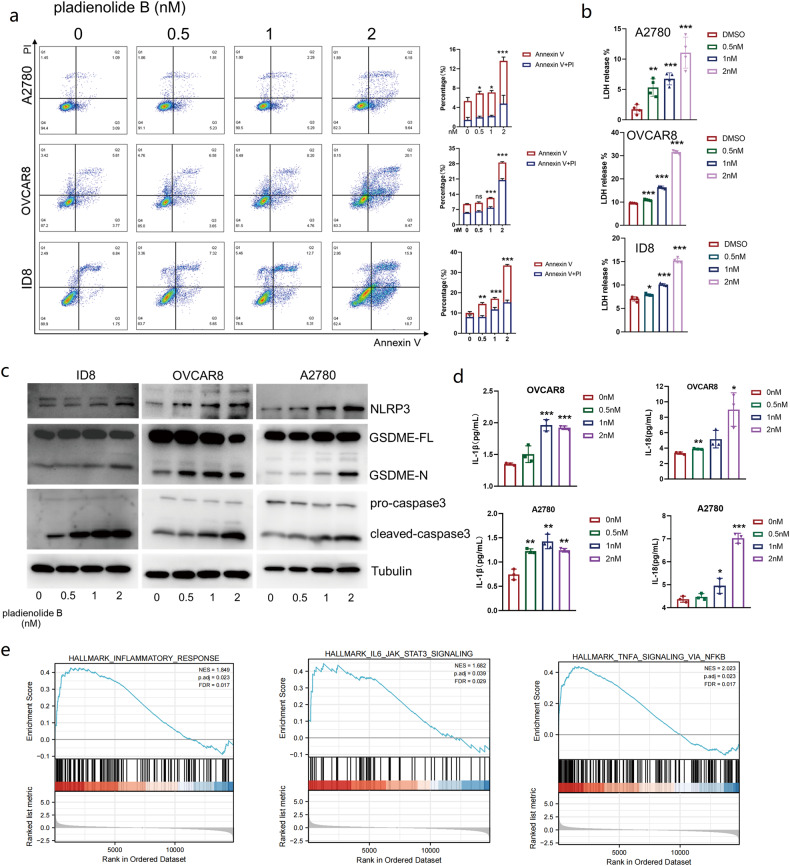
Pladienolide B induces ovarian cancer cell pyroptosis. **a** Flow cytometry of propidiumiodide (PI) and Annexin V-stained ovarian cancer cells after different concentrations of pladienolide B treatment for 72 h. (The statistical test for differences was compared with the 0 nM group.). **b** LDH releases of ovarian cancer cells were detected after different concentrations of pladienolide B treatment for 72 h. (The statistical test for differences was compared with the DMSO group.). **c** Biomarkers of pyroptosis were detected by western blot after different concentrations of pladienolide B treatment for 72 h. **d** Concentrations of IL-1β and IL-18 in cell culture supernatant after different concentrations of pladienolide B treatment for 72 h were analyzed by ELISA assay. (The statistical test for differences was compared with the 0 nM group.). **e** GSEA analysis of “HALLMARK-INFLAMMATORY-RESPONSE”, “HALLMARK-IL6-JAK-STAT3-SIGNALING”, “HALLMARK-TNFA-SIGNALING-VIA-NFKB” pathway from RNA-seq data of OVCAR8 cells treated with DMSO or pladienolide B for 72 h (*n* = 3 each group). *p* values were determined by One-Way ANOVA tests. *p < 0.05. **p < 0.01. ***p < 0.001. (All original blot images could be found in supplementary materials.).

Next, we characterized the functional consequences of inhibition SF3B1 in ovarian cancer. As expected, plate cloning and CCK8 assays showed that inhibition of SF3B1 by pladienolide B potently inhibited ovarian cancer cell growth, with an IC50 of 2–4 nM (Figure S2a, b). Annexin V and PI staining showed that pladienolide B induced cell death in a dose-dependent manner (Fig. 3a). Flow cytometry and western blotting were used to detect biomarkers of the different cell death pathways. As shown in Figure S2c, d, e, pharmacological inhibition of SF3B1 by pladienolide B induced cell death not through ferroptosis or necroptosis, but partly through apoptosis and mainly through pyroptosis. Release of LDH, which is one of the characteristics of pyroptosis, was detected in A2780, OVCAR8, and ID8 cells treated with different concentrations pladienolide B. As shown in Fig. 3b, pladienolide B exposure promoted LDH release in a dose-dependent manner. Moreover, genetic inhibition of SF3B1 by small interfering RNA (siRNA) induced cell death and LDH release (Figure S3a, b). Meanwhile, pladienolide B increased NLR family pyrin domain containing 3 (NLRP3), cleaved caspase3, and N-terminal GSDME expression (Fig. 3c), which are hallmarks of pyroptosis. IL-1β and IL-18 were inflammatory cytokines secreted by pyroptotic cells. ELISA assay showed that pladienolide B increased the concentrations of IL-1β and IL-18 in the cell culture supernatant (Fig. 3d). Genetic inhibition of SF3B1 consistently increased N-terminal GSDME expression and the concentrations of IL-1β and IL-18 in the cell culture supernatant (Figure S3c, d). Furthermore, inhibition of SF3B1 by pladienolide B consistently enriched pathways related to inflammasome activation such as inflammatory response, IL6-JAK-STAT3, and TNFA-NF-kB pathways (Fig. 3e). Taken together, the results suggested that inhibiton of SF3B1 induces pyroptosis of ovarian cancer cells.

### Inhibition of SF3B1 induces ovarian cancer cell pyroptosis through splicing regulation of BCL2L2

**Fig. 4:**
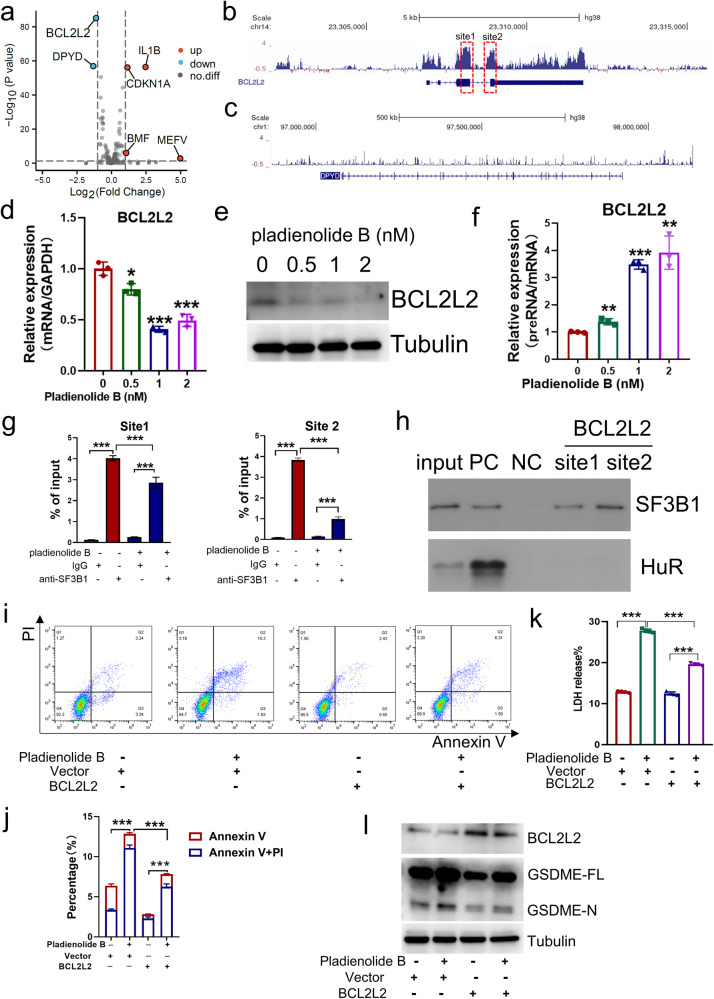
Targeted inhibition of SF3B1 by pladienolide B induces ovarian cancer cell pyroptosis partly through BCL2L2. **a** Volcano map of apoptosis and pyroptosis-associated genes from RNA-seq data of OVCAR8 cells treated with DMSO or pladienolide B for 72 h (*n* = 3 each group). **b**, **c** Binding peak of SF3B1 on BCL2L2 and DPYD from eCLIP-seq data of POSTAR3 database. (site 1 represents the junction between exon 3 and intron 3 of BCL2L2, while site 2 denotes the junction between intron 3 and exon 4 of BCL2L2). **d**, **e** Expression of BCL2L2 in OVCAR8 cell was detected by qPCR and western blot after different concentrations of pladienolide B treatment for 72 h. (The statistical test for differences was compared with the 0 nM group.). **f** The ratio of preRNA to mRNA of BCL2L2 was detected by qPCR in OVCAR8 cells after different concentrations of pladienolide B treatment for 72 h. (The statistical test for differences was compared with the 0 nM group.). **g** Enrichment of BCL2L2 transcripts in RIP samples from OVCAR8 cells with or without pladienolide B treatment using SF3B1 antibody or IgG was analyzed by qPCR. **h** Interaction of BCL2L2 transcripts and SF3B1 was verified by RNA-pulldown assay. PC means positive control, and NC means negative control. **I**, **j** Flow cytometry of propidiumiodide (PI) and Annexin V-stained OVCAR8 cells with or without pladienolide B treatment and BCL2L2 or control vector transfection. **k** LDH releases of OVCAR8 cells with or without pladienolide B treatment and BCL2L2 or control vector transfection were detected. **l** Expression of BCL2L2 and GSDME were detected by western blot in OVCAR8 cells with or without pladienolide B treatment and BCL2L2 or control vector transfection. *p* values were determined by One-Way ANOVA tests. *p < 0.05. **p < 0.01. ***p < 0.001. (All original blot images could be found in supplementary materials.).

In order to explore the mechanism of SF3B1 inhibition induced pyroptosis, we further analyzed the expression of genes related to apoptosis and pyroptosis based on the RNA-seq data of OVCAR8 cells treated with DMSO or pladienolide B because of the crosstalk between apoptosis and pyroptosis [28, 29]. The apoptosis- and pyroptosis-related genes were obtained from the MSigDB database. As shown in Fig. 4a, BCL2L2 and DPYD levels significantly decreased after pladienolide B treatment. Then binding peaks of SF3B1 on BCL2L2 and DPYD were analyzed using the POSTAR3 database. As shown in Fig. 4b, c, SF3B1 had distinct binding peaks for BCL2L2 but not DPYD. qPCR and western blotting results showed that exposure to pladienolide B reduced the expression of BCL2L2. In addition, correlation analysis of BCL2L2 and SF3B1 in TCGA cohort showed that the expression of BCL2L2 and SF3B1 was positively correlated (Figure S4a). Moreover, inhibition of SF3B1 by siRNA also reduced the expression of BCL2L2 (Figure S4b, c, d). Visualization of the exon junctions of BCL2L2 revealed a reduction in exon connectivity upon SF3B1 suppression (see Figure S4E). Analyses by rMATS indicate that inhibiting SF3B1 did not significantly alter the alternative splicing of BCL2L2 (A5SS, FDR = 1) (Table S4). This suggests that SF3B1 inhibition might decrease the splicing efficiency of BCL2L2. Next, we designed primers for pre-mRNA and mRNA of BCL2L2 (Figure S4f), and used the ratio of pre-mRNA to mRNA to represent the splicing efficiency. Then, qPCR was used to detect changes in BCL2L2 splicing efficiency following pladienolide B treatment or SF3B1 knockdown. As expected, inhibition of SF3B1 by pladienolide B or siRNA increased the ratio of BCL2L2 pre-mRNA to mRNA; that is, inhibition of SF3B1 decreased the splicing efficiency of BCL2L2 (Fig. 4f and Figure S4g). Furthermore, RIP-PCR results showed that the SF3B1 protein binds to the BCL2L2 transcript, and pladienolide B prevents the binding (Fig. 4g). RNA-pulldown was conducted to further confirm the binding of SF3B1 to BCL2L2; the results proved the binding of the BCL2L2 transcript to the SF3B1 protein (Fig. 4h).

To further explore whether the inhibition of SF3B1 induces pyroptosis through BCL2L2, rescue experiments were conducted. Annexin V and PI staining results showed that overexpression of BCL2L2 partially rescued the inhibition of SF3B1 by pladienolide B-induced cell death (Fig. 4i, j). The LDH release analysis results also showed that BCL2L2 overexpression partially rescued the inhibition of SF3B1 by pladienolide B-induced LDH release (Fig. 4k). Moreover, the increase in N-terminal GSDME expression by pladienolide B was also partly rescued by overexpression of BCL2L2 (Fig. 4l). In summary, we concluded that BCL2L2 is a direct target gene of SF3B1 and that the inhibition of SF3B1 induces ovarian cancer cell pyroptosis at least partly through BCL2L2.

### Antitumor effect of pladienolide B relies on macrophages

**Fig. 5:**
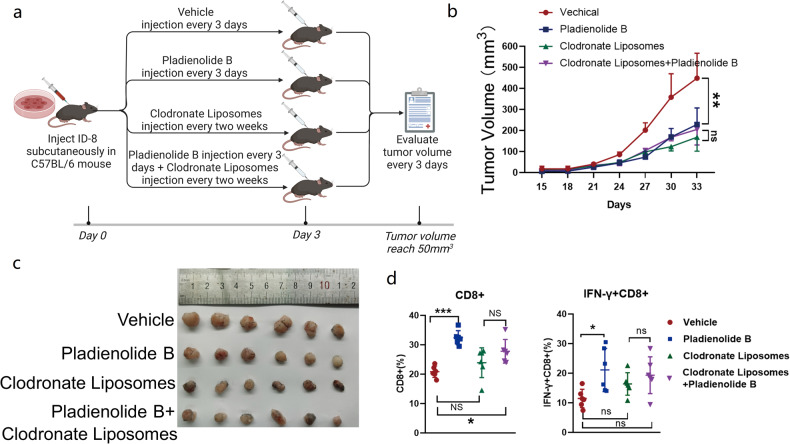
Macrophages are essential for the antitumor effects of pladienolide B. **a** Schematic diagram of tumor transplantation and drug administration in mice. ID8 cells (2 × 10^5^/mouse) were subcutaneously injected into the left groin of C56BL/6 mice (6–8 weeks old). After three days, mice were randomized into treatment cohorts accordingly (*n* = 6 for each group) and given drug treatment: vehicle (10% DMSO in PBS) or pladienolide B (0.5 mg/kg, every 3 days, 6 times) or clodronate liposomes (150 µL/mouse, every 2 weeks) or pladienolide B + clodronate liposomes. After the tumors were palpable, tumor volume was evaluated every 3 days. Pladienolide B treatment was stopped at day 18 and clodronate liposomes exposure was continued. Then tumor growth was kept to be observed for another 2 weeks. Clodronate liposomes were used to clear macrophage. **b** Growth curve of tumor volume. **c** Image of transplantation tumors. **d** The proportion of CD8+ cells to CD3+ cells and the proportion of IFN-γ + CD8+ cells to CD8+ cells. *p* values were determined by One-Way ANOVA tests. *p < 0.05. **p < 0.01. ***p < 0.001.

Pyroptosis is immunogenic death accompanied by large amounts of cellular contents release [30, 31]. We speculated that cellular contents released by pyroptotic cells may activate antitumor immune cells. Three major types of cytotoxic immune cells, macrophages, T cells, and NK cells, were used to test the effect of the pladienolide B-treated cell culture supernatant and individual pladienolide B treatments on their cytotoxicity (Figure S5a). Compared with T cells and NK cells, bone marrow-derived macrophages (BMDMs) showed the most obvious increase in cytotoxicity after treatment with pladienolide B-treated cell culture supernatant (Figure S5b). These results suggest that macrophages may be critical in the pladienolide B-induced slowing of tumor growth and improvement of the tumor immune microenvironment. Clodronate liposomes were used to eliminate macrophages in C57BL/6 mice to test the effect of macrophages on the antitumor effect of pladienolide B (Fig. 5a). Interestingly, macrophage depletion significantly reduced tumor volume in mice, as expected, but eliminated the antitumor effect of pladienolide B (Fig. 5b, c). Moreover, although macrophage depletion tended to increase the proportion of CD8+ and IFN-γ + CD8 + T cells, the increase was not significant, and the depletion of macrophages partly removed the pladienolide B-induced increase of CD8+ and IFN-γ + CD8 + T cell proportion among tumor-infiltrating lymphocytes (Fig. 5d). Overall, we concluded that pladienolide B-treated ovarian cancer cell supernatant increased macrophage cytotoxicity, which was essential for the antitumor effects of pladienolide B.

### Pladienolide B activated macrophages through mtDNA-cGAS-STING pathway

**Fig. 6:**
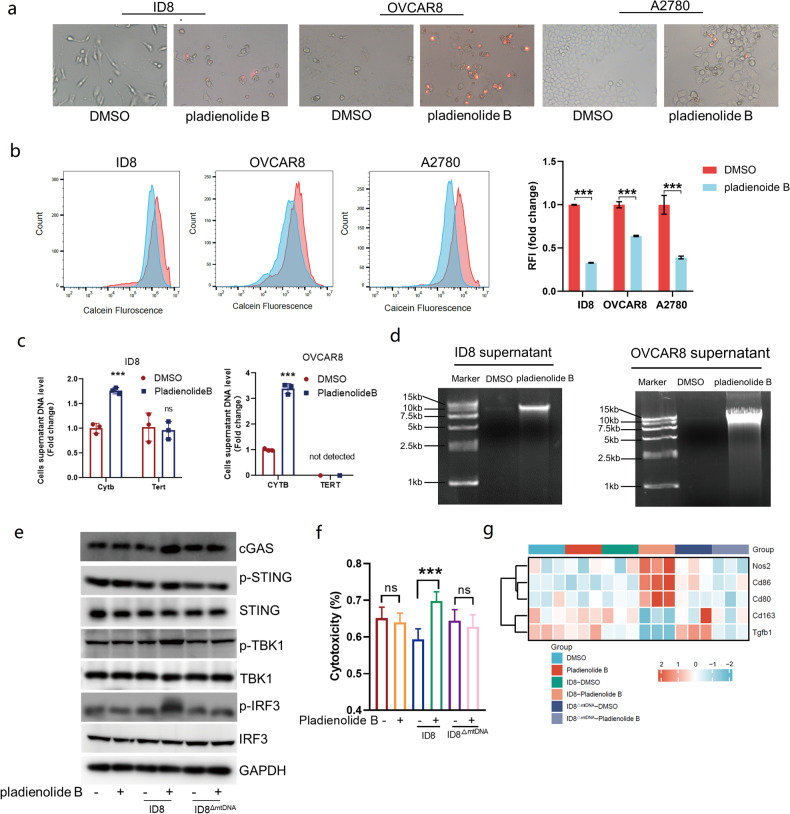
Pladienolide B activated macrophages through mtDNA-cGAS-STING pathway. **a** Ovarian cancer cells treated by DMSO or pladienolide B were stained by PI. **b** The openness of mitochondrial permeability transition pores (mPTP) was measured by fluorescent probe Calcein and flow cytometry. The stronger the Calcein fluorescence, the lower the degree of opening, and the weaker the Calcein fluorescence, the higher the degree of opening. **c** Mitochondrial and nuclear DNA in culture supernatant of ID8 and OVCAR8 cells treated with DMSO or pladienolide B were detected by qPCR. **d** DNA in culture supernatant of ID8 and OVCAR8 cells treated by DMSO or pladienolide B were extracted and detected by agarose gel electrophoresis. **e** cGAS-STING pathway in BMDM treated by DMSO or pladienolide B, or supernatant of ID8 treated by DMSO or pladienolide B, or supernatant of ID8^△mtDNA^ treated by DMSO or pladienolide B were analyzed by western blot. **f** Cytotoxicity of BMDM treated by DMSO or pladienolide B, or supernatant of ID8 treated by DMSO or pladienolide B, or supernatant of ID8^△mtDNA^ treated by DMSO or pladienolide B were analyzed by co-culture of BMDM and ID8-LUC with 10:1 effect target ratio. **g** Heatmap of Nos2, Cd86, Cd80, Cd163, and Tgfb1 expression detected by qPCR in mouse BMDM cells treated by DMSO or pladienolide B, or supernatant of ID8 treated by DMSO or pladienolide B, or supernatant of ID8^△mtDNA^ treated by DMSO or pladienolide B. *p* values were determined by Student t tests and One-Way ANOVA tests. *p < 0.05. **p < 0.01. ***p < 0.001. (All original gel and blot images could be found in supplementary materials.).

mtDNA is often released extracellularly by pyroptotic cells and can be engulfed by macrophages [32, 33]. To explore whether pladienolide B activates macrophages through mtDNA, we first evaluated cell membrane permeability following pladienolide B treatment using PI staining. As shown in Fig. 6a, pladienolide B increased cell membrane permeability of ID8, OVCAR8, and A2780 cells. Openness of mitochondrial permeability transition pores (mPTP) was measured using calcein staining. Flow cytometry results showed that pladienolide B increased the openness of the mPTP in ID8, OVCAR8, and A2780 cells (Fig. 6b). Moreover, qPCR results showed that pladienolide B increased the mtDNA levels, but not those of nuclear DNA, in ID8 and OVCAR8 cell supernatants (Fig. 6c). Agarose gel electrophoresis results showed that DNA in the supernatant of ID8 and OVCAR8 cells treated with pladienolide B was significantly enriched at the mtDNA position (Fig. 6d). Thus, we concluded that pladienolide B induces the release of mtDNA from ovarian cancer cells.

To further explore the effect of mtDNA on macrophages, cy3-dCTP-labeled mtDNA was incubated with BMDMs. As shown in the immunofluorescence images in Figure S6a, BMDMs engulfed mtDNA. In addition, mtDNA activated the cGAS-STING pathway and increased the BMDM cytotoxicity (Figure S6b, c). To confirm whether the supernatants from pladienolide B-treated ovarian cancer cells activated macrophages through mtDNA, mtDNA-deleted ID8 cells (ID8^△mtDNA^) were established by three-week exposure to ethidium bromide (Figure S6d). As shown in Figure S6e, f, mtDNA deletion in ID8 cells did not prevent pladienolide B-induced pyroptosis, but prevented an increase in mtDNA levels in the supernatants of pladienolide B-treated cells. Pladienolide B alone, supernatants of ID8 cells treated with pladienolide B, supernatants of ID8^△mtDNA^ treated with pladienolide B, and the corresponding controls were co-cultured with BMDMs for 24 h. As expected, the supernatant of ID8 cells treated with pladienolide B, but not pladienolide B alone or the supernatant of ID8^△mtDNA^ cells treated with pladienolide B activated the cGAS-STING pathway and increased the cytotoxicity of BMDMs (Fig. 6e, f). In addition, compared to pladienolide B alone and the supernatant of ID8^△mtDNA^ cells treated with pladienolide B, the supernatant of ID8 cells treated with pladienolide B increased the expression of Nos2, Cd86, and Cd80, and decreased that of Cd163 and Tgfb1 in BMDMs (Fig. 6g). Taken together, the results suggested that pladienolide B induces mtDNA release in ovarian cancer cells, and macrophages engulf mtDNA and become activated through mtDNA-cGAS-STING pathway.

### Pladienolide B augments the antitumor effects of αPDL1 in ovarian cancer via immune activation

**Fig. 7:**
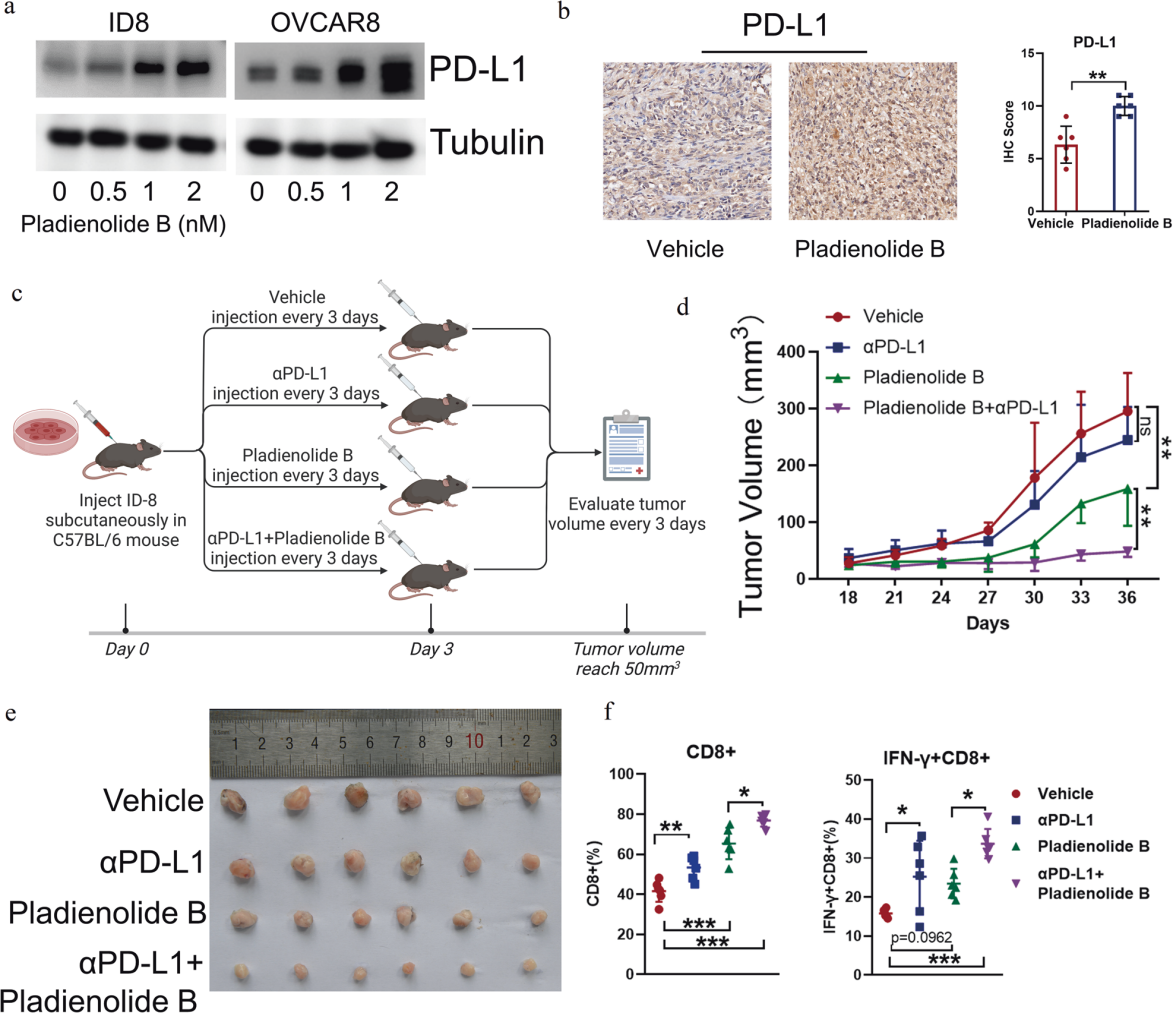
Pladienolide B enhances the antitumor effect of αPDL1. **a** Expression of PD-L1 in OVCAR8 and ID8 cells treated with different concentration of pladienolide B was detected by western blot. **b** Expression of PD-L1 in mice transplant tumor of vehicle group and pladienolide B group in Fig. 2c was analyzed by immunohistochemistry staining. **c** Schematic diagram of tumor allotransplantation and drug administration in mice. ID8 cells (2 × 10^5^/mouse) were subcutaneously injected into the left groin of C56BL/6 mice (6–8 weeks old). After three days, mice were randomized into treatment cohorts accordingly (*n* = 6 for each group) and given drug treatment: vehicle (10% DMSO in PBS and IgG isotype control), αPDL1 (200 µg/ mouse, every 3 days, 6 times), pladienolide B (0.5 mg/kg, every 3 days, 6 times) and αPDL1+pladienolide B. After the tumors were palpable, tumor volume was evaluated every 3 days. Drug administration was stopped at day 18 and tumors growth was kept to be observed for another 18 days. **d** Growth curve of tumor volume. **e** Image of transplantation tumors. **f** The proportion of CD8+ cells to CD3+ cells and the proportion of IFN-γ + CD8+ cells to CD8+ cells. *p* values were determined by One-Way ANOVA tests. *p < 0.05. **p < 0.01. ***p < 0.001. (All original blots images could be found in supplementary materials.).

αPDL1 is a widely used immune checkpoint inhibitor for cancer therapy but has a low response rate in ovarian cancer. Since pladienolide B treatment increased cytotoxic lymphocyte infiltration (Fig. 2f–i), we sought to determine whether it affects the antitumor effect of αPDL1. Western blotting results showed that pladienolide B increased the expression of PD-L1 in OVCAR8 and ID8 cells (Fig. 7a). Immunohistochemical staining showed that pladienolide B treatment increased the expression of PD-L1 in mouse tumors (Fig. 7b). ID8 cells were injected into C57BL/6 mice, and pladienolide B alone, αPDL1 alone, or a combination of the two was administered to the mice for treatment (Fig. 7c). αPDL1 alone had no significant effect on tumor growth, while pladienolide B alone decreased tumor growth. Moreover, the combination of pladienolide B and αPDL1 effectively augmented the decrease in tumor burden (Fig. 7d, e). As shown in Fig. 7f, αPDL1 alone or pladienolide B alone increased the population of CD8^+^ and IFN-γ^+^CD8^+^ T cells, but the combination increased them more significantly.

## Discussion

Immunotherapy is one of the most promising treatments for malignant tumors in recent years and has demonstrated encouraging efficacy in various solid tumors. However, its effectiveness in ovarian cancer has been disappointing, possibly due to factors that directly or indirectly inhibit the CTL response. There is evidence to suggest that combining immunotherapy with targeted drugs can enhance its efficacy in treating ovarian cancer, possibly by improving the tumor immune microenvironment. Recent research suggests that PARP inhibitors can activate the STING pathway in tumor cells, leading to increased T-cell infiltration and improved efficacy of immunotherapy in ovarian cancer [34]. These findings have been corroborated by promising response rates observed in the TOPACIO/KEYNOTE-162 (phase I-II) and MEDIOLA trials, where the combination of immune checkpoint inhibitors (ICIs) and PARP inhibitors was employed [35, 36]. Refining the immune microenvironment in ovarian cancer is increasingly recognized as a critical determinant of successful outcomes from immunotherapy. Based on this, we identified SF3B1, a splicing factor that is highly expressed in ovarian cancer and negatively correlated with prognosis and cytotoxic lymphocyte infiltration, through screening TCGA and CPTAC databases and validating with clinical tissue microarrays.

SF3B1 plays a crucial role in various malignant tumors as a splicing factor, and there has been a long-standing exploration of small-molecule splicing modulators to SF3b as potential anti-cancer therapeutics [37, 38]. Recently, studies have highlighted the significance of spliceosome-targeted therapies in cancer once again [39, 40]. Bowling et al. found that in MYC-driven triple-negative breast cancer, spliceosome-targeted therapy causes widespread cytoplasmic accumulation of mis-spliced dsRNA and triggers antiviral signaling and extrinsic apoptosis, which is closely related to the immune [41]. While in our study, targeting the spliceosome by pladienolide B caused pyroptosis in non-MYC-driven instead of dsRNA accumulation.

Inducing pyroptosis is a promising strategy for cancer treatment [42]. Pyroptosis modulates various immune cells in the tumor immune microenvironment, inflammatory damage-associated molecular patterns (DAMPs), such as ATP, HMGB1, and mtDNA, released by pyroptotic cells, play an important role in host defense [43, 44]. IL-18 is a key activator of NK and Th1 cells and forms a positive loop with interferon-γ (IFN-γ) [45, 46]. HMGB1 leads to the activation of macrophages and TNF secretion and induces the infiltration of CTLs [47, 48]. In our study, NK cells, T cells, and BMDMs were exposed to the supernatant of pyroptotic cells and their cytotoxicity to tumor cells was detected. We found that the supernatant of pyroptotic cells increased the cytotoxicity of BMDMs, but not of NK or T cells. NK and T cells originating from the spleen rather than the tumor tissue may partly explain these results. The key finding of our study is that the release of mtDNA from pyroptotic tumor cells is crucial for the activation of macrophages, and we also identified BCL2L2 as a SF3B1 target gene that plays a role in pyroptosis.

BCL2L2 is well-known as an anti-apoptotic protein of the BCL2 family [49], but recent studies have shown that it also plays a crucial role in cell pyroptosis. Bcl-2 is closely related to caspase-3 and can directly inhibit the cleavage of GSDMD mediated by caspase-1, thereby reducing the incidence of cell pyroptosis and the release of IL-1β [28, 50]. Although caspase-3 is not unique to pyroptosis, gasdermins are the core switch that leads to the transition from apoptosis to pyroptosis [43, 44].

TAMs play various roles in tumor progression. M1 macrophages have the capacity to kill and remove tumor cells, whereas M2 macrophages promote tumor propagation [51, 52]. Depletion of macrophages by clodronate liposomes significantly reduced the tumor burden of the ID8 graft but was not sufficient to clear tumors in our study. We found that exposure to the supernatant of pladienolide B-treated cells increased the cytotoxicity of BMDMs, increased the expression of M1 macrophage markers, and reduced the expression of M2 macrophage markers in vitro. More importantly, pladienolide B showed no antitumor effect in macrophage-depleted mice, indicating that pladienolide B exerts its antitumor effect relying on the presence of macrophages.

Our results suggest that SF3B1 is highly expressed in ovarian cancer and is associated with poor prognosis and low cytotoxic immune cell infiltration. Inhibition of SF3B1 triggers pyroptosis in ovarian cancer cells and promotes infiltration of cytotoxic lymphocytes. Macrophages engulf mtDNA released by pyroptotic cells and play an important role in the antitumor effect of pladienolide B. Moreover, pladienolide B increases the expression of PD-L1 and synergizes with ICB therapy in ovarian cancer. Our study provided preclinical evidence for SF3B1 inhibition and ICB combination therapy in ovarian cancer. Nevertheless, our study’s findings are confined to animal models of ovarian cancer in mice and further validation using patient-derived xenograft models is yet to be undertaken.

Inhibition of SF3B1 induces ovarian cancer cell pyroptosis by splicing regulation of BCL2L2, resulting in the release of mtDNA. Macrophages engulfed mtDNA and polarized towards M1 through mtDNA-cGAS-STING pathway, thereby improving the immune microenvironment of ovarian cancer and enhancing the therapeutic effect of αPDL1. Therefore, SF3B1 inhibitor holds significant potential in combination with ICB therapy of ovarian cancer.

### Data sharing statement

The datasets supporting the conclusions of this article are included within the article and its additional files. The datasets used and analyzed during the current study are available from the corresponding author on reasonable request. The RNA-seq raw data generated in this study have been deposited in the NCBI GEO database under the accession number GSE222199.

### Supplementary information

A reproducibility checklist

Supplementary figure legends

Supplementary tables

Table S4

Original western blots

## Data Availability

Protein expression data was downloaded from CPTAC Assay Portal (http://assays.cancer.gov/). RNA expression data were obtained from the TCGA (https://portal.gdc.cancer.gov/) databases. Prognostic analysis was performed by an online Kaplan–Meier plotter database (http://kmplot.com/analysis/) [26]. SF3B1 eCLIP binding peak was visualized with online POSTAR3 database (http://111.198.139.65/index.html) [27]. Other data used during the current study were available from the corresponding author on reasonable request.
