# Supplementary material for: Inhibition of SF3B1 improves the immune microenvironment through pyroptosis and synergizes with αPDL1 in ovarian cancer
Source: Cell Death Dis. 2023 Nov 27;14(11):775. doi: 10.1038/s41419-023-06301-1 (PMC10682409; doi:10.1038/s41419-023-06301-1)
Supplement: Supplementary file 1 — A reproducibility checklist [file 41419_2023_6301_MOESM1_ESM.pdf]

Corresponding Author Name:_Kun Song, Chaoyang Sun and Beihua
Manuscript Number:__CDDIS-23-2236R___

SPRINGER NATURE

Reporting Summary

Springer Nature wishes to improve the reproducibility of the work that we publish. This checklist is used to ensure good reporting standards and to improve the reproducibility. Please respond completely to all questions relevant to your manuscript. For more information, please read the journal's Guide to Authors.

☑ **Check here to confirm that the following information is available in the Material & Methods section:**

- **The exact sample size (*n*)** for each experimental group/condition, given as a number, not a range
- **A description of the sample collection** allowing the reader to understand whether the samples represent technical or biological replicates (including how many animals, litters, culture, etc.)
- **A statement of how many times the experiment shown was replicated in the laboratory**
- **Definitions of statistical methods and measures**: For small sample sizes (n<5) descriptive statistics are not appropriate, instead plot individual data points
  - Very common tests, such as *t*-test, simple χ^2 tests, Wilcoxon and Mann-Whitney tests, can be unambiguously identified by name only, but more complex techniques should be described in the methods section
  - Are tests one-sided or two-sided?
  - Are there adjustments for multiple comparisons?
  - **Statistical test results**, e.g., ***P* values**
  - Definition of **'center values'** as **median or mean**;
  - Definition of **error bars as s.d. or s.e.m. or c.i.**

Please ensure that the answers to the following questions are reported in the manuscript itself. We encourage you to include a specific subsection in the methods section for statistics, reagents and animal models. Below, provide the page number or section and paragraph number.

Statistics and general methods	Reported in section/paragraph or page #
1. How was the sample size chosen to ensure adequate power to detect a pre-specified effect size? (Give section/paragraph or page #)	We used tissue microarray samples (obtained from 261 patients with complete clinical information who had been treated at Qilu Hospital of Shandong University) to analyse by IHC.(Reported in section of Methods - Immunohistochemical staining (IHC))
For animal studies, include a statement about sample size estimate even if no statistical methods were used.	Reported in section of Methods - ID8 mouse tumor model
2. Describe inclusion/exclusion criteria if samples or animals were excluded from the analysis. Were the criteria pre-established? (Give section/paragraph or page #)	Reported in section of Methods - Immunohistochemical staining (IHC).<br>Criteria was pre-established.
3. If a method of randomization was used to determine how samples/animals were allocated to experimental groups and processed, describe it. (Give section/paragraph or page #)	We used the Block Randomization method to allocate the mices to the groups.
For animal studies, include a statement about randomization even if no randomization was used.	We stated that we used randomized methods in animal experiments. It is reported in section of Methods-ID8 mouse tumor model.

4. If the investigator was blinded to the group allocation during the experiment and/or when assessing the outcome, state the extent of blinding. (Give section/paragraph or page #)	When the experimental results were collected, the investigator was absolutely blind to prevent the occurrence of deviation. It is reported in section of Methods- Immunohistochemical staining (IHC).
For animal studies, include a statement about blinding even if no blinding was done.	We stated that the investigator was absolutely blind for animal studies.
5. For every figure, are statistical tests justified as appropriate?	Yes
Do the data meet the assumptions of the tests (e.g., normal distribution)?	Yes
Is there an estimate of variation within each group of data?	Yes
Is the variance similar between the groups that are being statistically compared? (Give section/paragraph or page #)	There was no significant difference in variances between the groups. It is reported in section of Methods -Statistical analysis.

Reagents

	**Reported in section/paragraph or page #**
6. Report the source of antibodies (vendor and catalog number)	Reported in section of Method - Antibodies and compounds and Supplementary Tables - Table S1 Information of antibodies used in the study.
7. Identify the source of cell lines and report if they were recently authenticated (e.g., by STR profiling) and tested for mycoplasma contamination	Cells were purchased from the regular company, and we described it in section of Methods - Cell lines and cell culture.

Animal Models

	**Reported in section/paragraph or page #**
8. Report species, strain, sex and age of animals	C57BL/6 mice, female, 6-8-weeks-old
9. For experiments involving live vertebrates, include a statement of compliance with ethical regulations and identify the committee(s) approving the experiments.	We have got the approval from Animal Experiment Ethics Committee of Qilu Hospital, which is reported in section of Methods - ID8 mouse tumor model.

10. We recommend consulting the ARRIVE guidelines (*PLoS Biol.* **8**(6), e1000412,2010) to ensure that other relevant aspects of animal studies are adequately reported.

Human subjects

	Reported in section/paragraph or page #
11. Identify the committee(s) approving the study protocol.	Reported in section of Declaration: the Animal Experiment Ethics Committee of Qilu Hospital.
12. Include a statement confirming that informed consent was obtained from all subjects.	Yes, it is reported in section of Immunohistochemical staining (IHC).
13. For publication of patient photos, include a statement confirming that consent to publish was obtained.	n/a<br>There are no patient photos in the manuscript.
14. Report the clinical trial registration number (at ClinicalTrials.gov or equivalent).	n/a<br>This study does not include a clinical trial.

15. For phase II and III randomized controlled trials, please refer to the CONSORT statement and submit the CONSORT checklist with your submission.

16. For tumor marker prognostic studies, we recommend that you follow the REMARK reporting guidelines.

Data deposition

	Reported in section/paragraph or page #
17. Provide accession codes for deposited data. Data deposition in a public repository is mandatory for:<br>a. Protein, DNA and RNA sequences<br>b. Macromolecular structures<br>c. Crystallographic data for small molecules<br>d. Microarray data	They are reported in section of Data sharing statement. RNA sequencing data was upload to GEO (GSE222199), and now in Private Status. We can always open access upon request of reviewers or editors.

Deposition is strongly recommended for many other datasets for which structured public repositories exist; more details on our data policy are available in the Guide to Authors. We encourage the provision of other source data in supplementary information or in unstructured repositories such as Figshare and Dryad. We encourage publication of Data Descriptors (see Scientific Data) to maximize data reuse.

18. If computer code was used to generate results that are central to the paper's conclusions, include a statement in the Methods section under "**Code availability**" to indicate whether and how the code can be accessed. Include version information as necessary and any restrictions on availability.	We think that our conclusions were derived mainly from cell functional experiments, animal experiments and clinical samples. Computer code was a confirmatory experiment and did not generate the central results.
