# Supplementary material for: Inhibition of SF3B1 improves the immune microenvironment through pyroptosis and synergizes with αPDL1 in ovarian cancer
Source: Cell Death Dis. 2023 Nov 27;14(11):775. doi: 10.1038/s41419-023-06301-1 (PMC10682409; doi:10.1038/s41419-023-06301-1)
Supplement: Supplementary file 2 — Supplementary figure legends [file 41419_2023_6301_MOESM2_ESM.doc]

# Inhibition of SF3B1 improves the immune microenvironment through pyroptosis and synergizes with αPDL1 in ovarian cancer

**Authors：**Shourong Wang^#1,2^, Yao Liu^#1,2^, Huimin Xiao^1,2^, Zhongshao Chen^1,2^, Xiaohang Yang^3^, Jingjing Yin^3^, Yingwei Li^1,2^, Cunzhong Yuan^1,2^, Shi Yan^1,2^, Beihua Kong*^1,2^，Chaoyang Sun*^3^, Kun Song*^1,2^

**#** The authors contribute equally

1. Department of Obstetrics and Gynecology, Qilu Hospital of Shandong University, Jinan, 250012, China.
2. Gynecologic Oncology Key Laboratory of Shandong Province, Qilu Hospital of Shandong University, Jinan, 250012, China.
3. Department of Obstetrics and Gynecology, Tongji Hospital, Tongji Medical College, Huazhong University of Science and Technology, Wuhan 430030, China.

*Corresponding Author:

Beihua Kong:[**kongbeihua@sdu.edu.cn**](mailto:kongbeihua@126.com)

Chaoyang Sun:**suncydoctor@gmail.com**

Kun Song:[**songkun2001226@sdu.edu.cn**](mailto:songkun2001226@sdu.edu.cn)

## Supplementary Figures

**
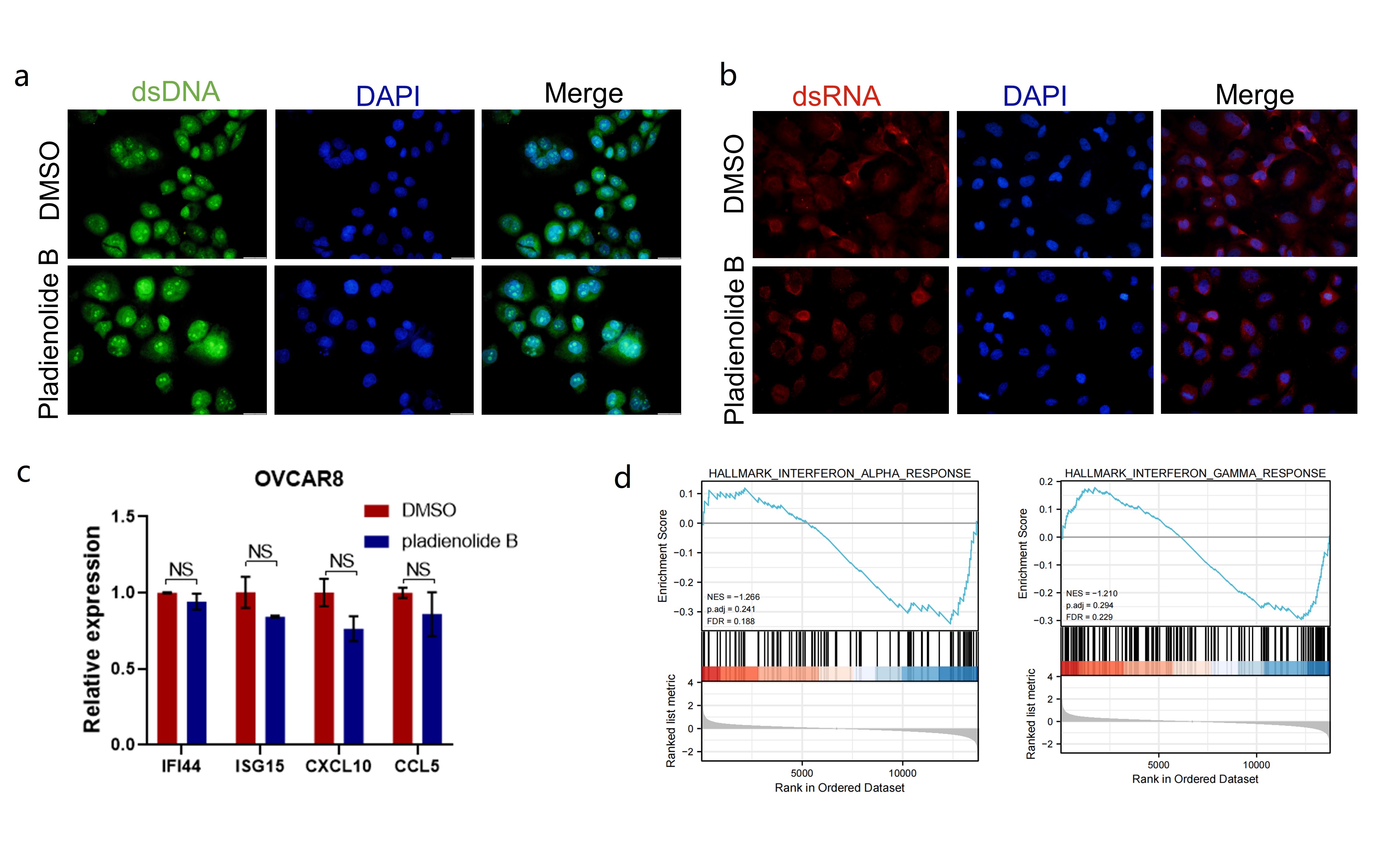
**

### Figure S1. Targeted inhibition of SF3B1 by pladienolide B fails to activate the interferon pathway.

a-b. Fluorescent staining of double-stranded DNA (A) and double-stranded RNA (B) in OVCAR8 cells with DMSO or pladienolide B treatment for 72h.

c. Quantification of interferon-stimulated gene by qPCR in OVCAR8 cells after with DMSO or pladienolide B treatment for 72h. Three independent experiments were shown.

d. GSEA analysis of “HALLMARK-INTERFERON-ALPHA-RESPONSE” and “HALLMARK-INTERFERON-GAMMA-RESPONSE” pathway from RNA-seq data of OVCAR8 cells treated with DMSO or pladienolide B for 72h (n=3 each group).


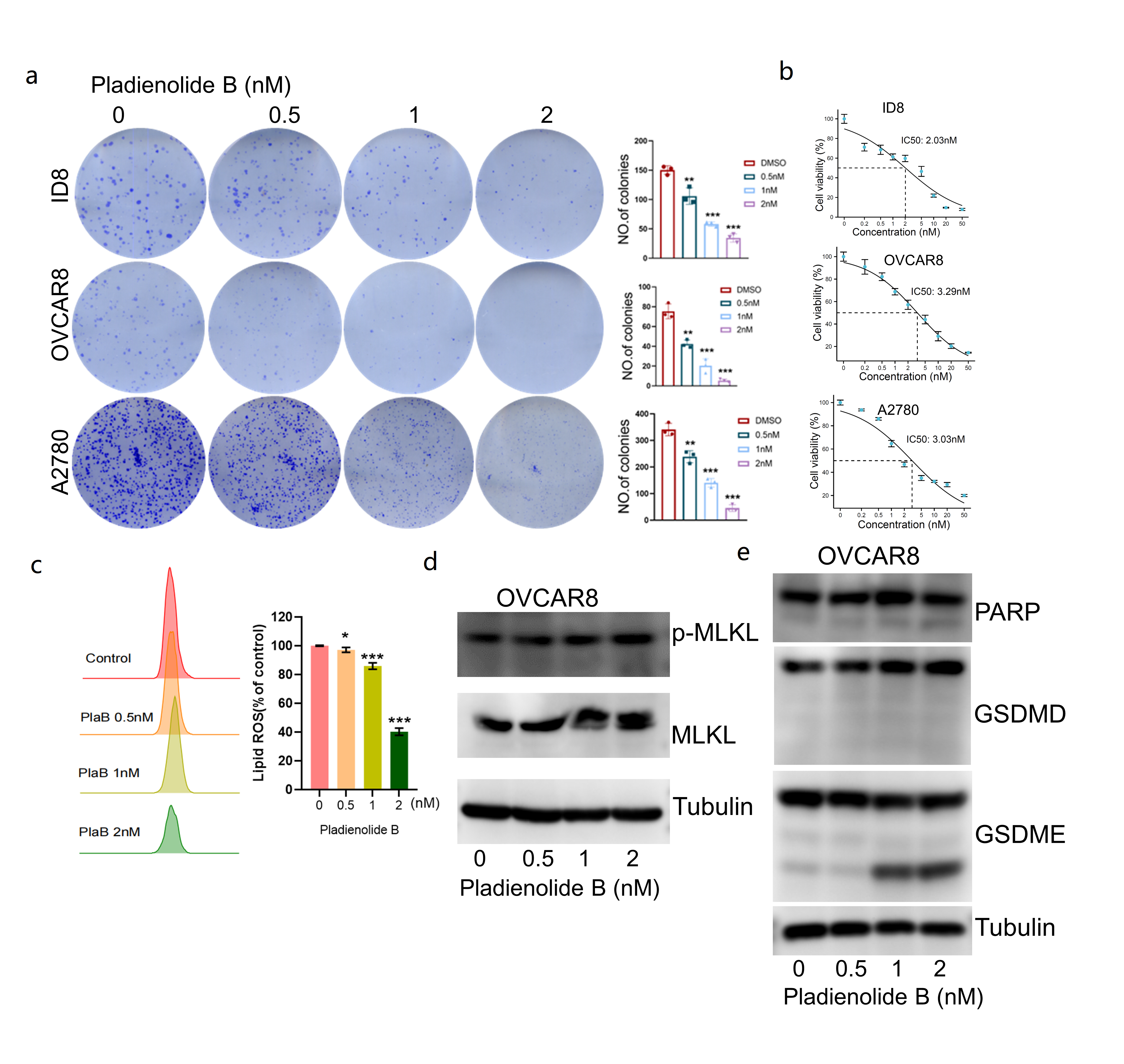


### Figure S2. Targeted inhibition of SF3B1 by pladienolide B inhibits cell growth and induces cell death.

a.Representative and statistical clonogenic picture of ID8, OVCAR8 and A27890 cells for different concentrations of pladienolide B treatment.

b.Cell viability of ID8, OVCAR8 and A27890 cells was measured by CCK8 assay after different concentrations of pladienolide B treatment for 72h. (The statistical test for differences was compared with the DMSO group.)

c. Lipid peroxides in OVCAR8 cells was detected by flow cytometry after treated with different concentrations of pladienolide B for 72h. (The statistical test for differences was compared with the 0nM group.)

d-e. Markers of necroptosis, apoptosis and pyroptosis were detected by western blotting. p values were determined by One-Way ANOVA tests. *p < 0.05. **p < 0.01. ***p < 0.001.

(All original blots images could be found in supplementary materials.)

**
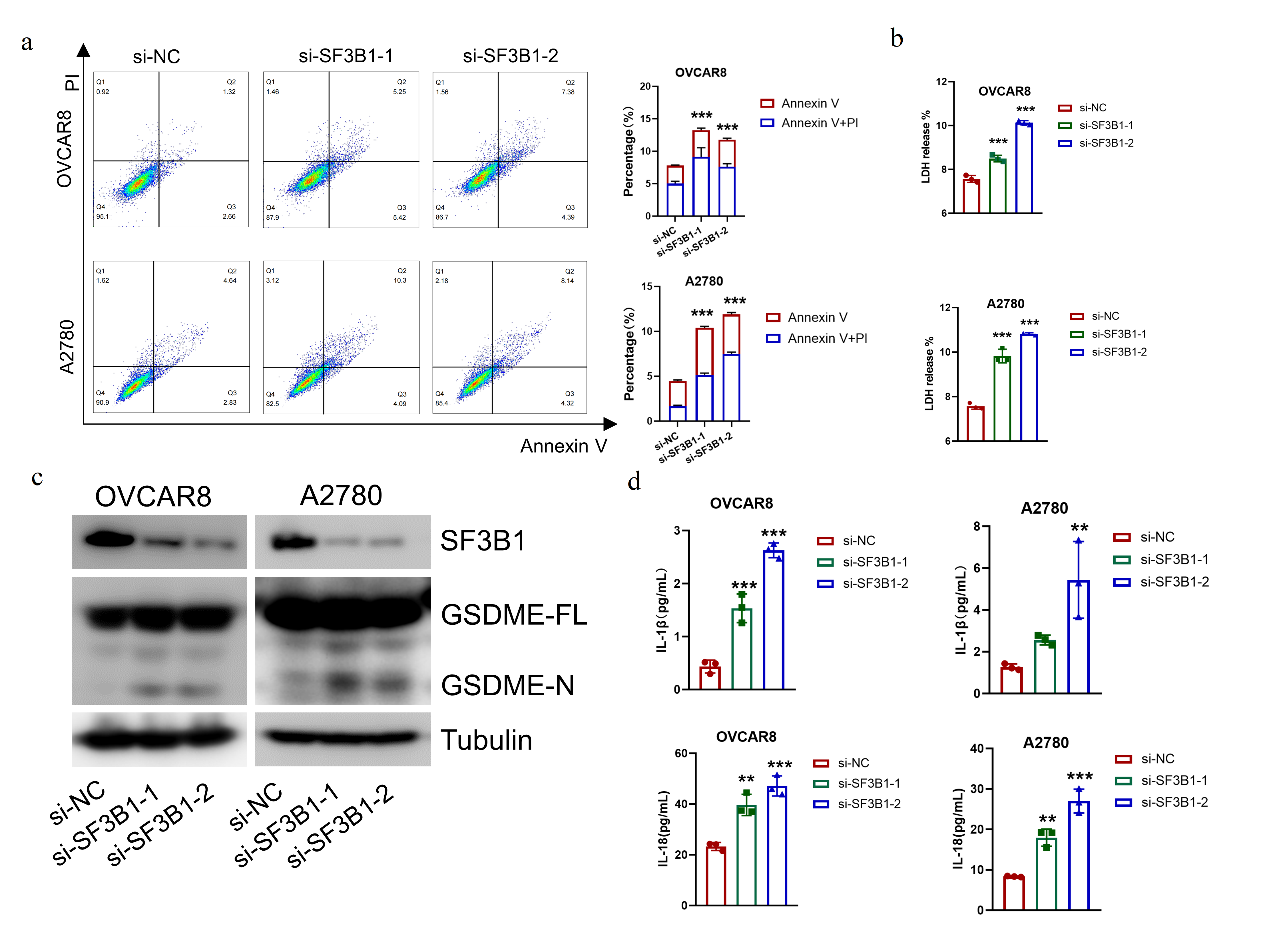
**

### Figure S3. Knock down of SF3B1 induces ovarian cancer cell pyroptosis.

1. Flow cytometry of propidiumiodide (PI) and Annexin V-stained ovarian cancer cells with SF3B1 or control siRNA transfection. (The statistical test for differences was compared with the si-NC group.)
2. LDH releases of ovarian cancer cells were detected after SF3B1 or control siRNA transfection for 72h. (The statistical test for differences was compared with the si-NC group.)
3. GSDME and SF3B1 expression were detected by western blot in OVCAR8 and A2780 cells with SF3B1 or control siRNA transfection.
4. Concentrations of IL-1β and IL-18 in OVCAR8 and A2780 culture supernatant with SF3B1 or control siRNA transfection were analyzed by ELISA assay. (The statistical test for differences was compared with the si-NC group.)

p values were determined by One-Way ANOVA tests. *p < 0.05. **p < 0.01. ***p < 0.001.

(All original blots images could be found in supplementary materials.)


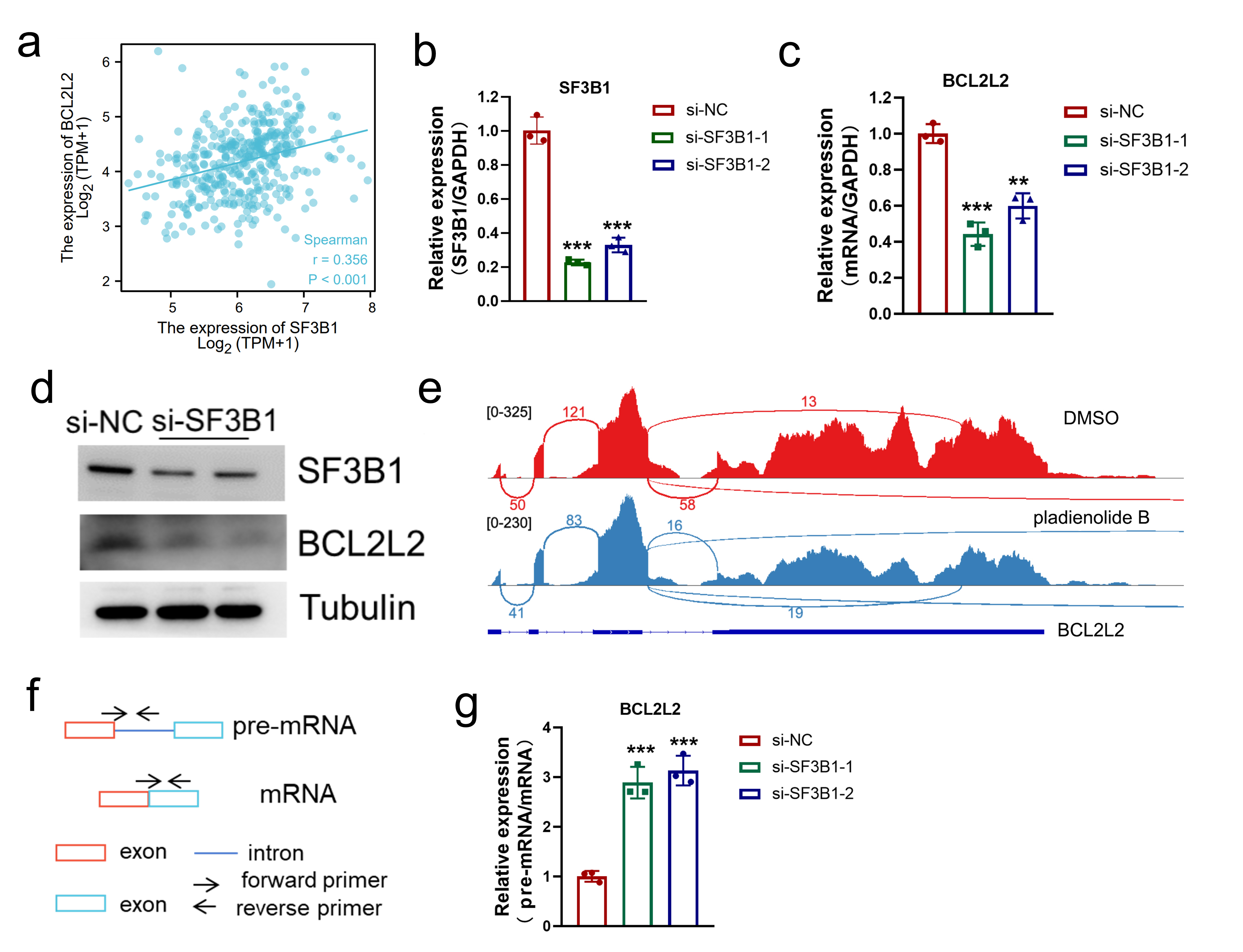


### Figure S4. SF3B1 regulates BCL2L2 expression.

1. Correlation between SF3B1 and BCL2L2 RNA expression in TCGA cohort.

b-c. RNA expression of SF3B1 and BCL2L2 was analyzed by qPCR in OVCAR8 cells with SF3B1 siRNA transfection. (The statistical test for differences was compared with the si-NC group.)

d. Protein expression of SF3B1 and BCL2L2 was detected by western blot in OVCAR8 cells with SF3B1 siRNA transfection.

e. Sashimi plot generated by Integrative Genomics Viewer (Version 2.16.2) showing RNA-seq mapping to BCL2L2 in OVCAR8 cells following exposure to plaB.

f. Schematic diagram of primers for BCL2L2 pre-mRNA and mRNA.Primers for pre-mRNA span intron-exon junctions and primers for mRNA span exon-exon junctions.

g. The ratio of preRNA to mRNA of BCL2L2 was detected by qPCR in OVCAR8 cells with SF3B1 siRNA transfection. (The statistical test for differences was compared with the si-NC group.)

p values were determined by One-Way ANOVA tests. *p < 0.05. **p < 0.01. ***p < 0.001.

(All original blots images could be found in supplementary materials.)


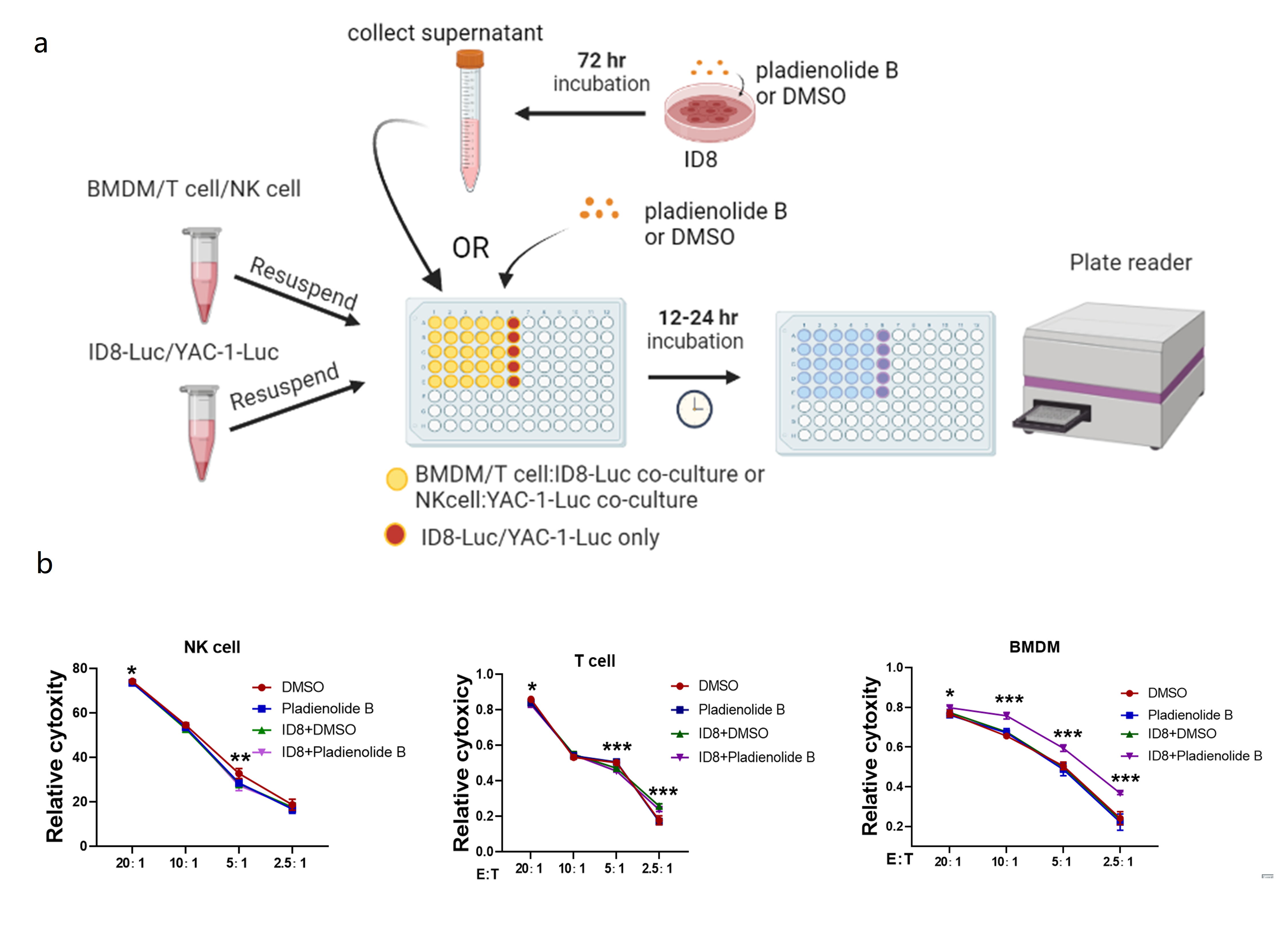


### Figure S5. Culture supernatant of pladienolide B-treated ID8 cells enhances cytotoxicity of BMDM.

1. Schematic diagram of cytotoxicity detection of NK cells, T cells, and BMDM.
2. Effect of pladienolide B only or supernatant of pladienolide B-treated ID8 cells on cytoxicity of NK cells, T cells and BMDM was detected. (The statistical test for differences was compared among all groups.)

p values were determined by One-Way ANOVA tests. *p < 0.05. **p < 0.01. ***p < 0.001.


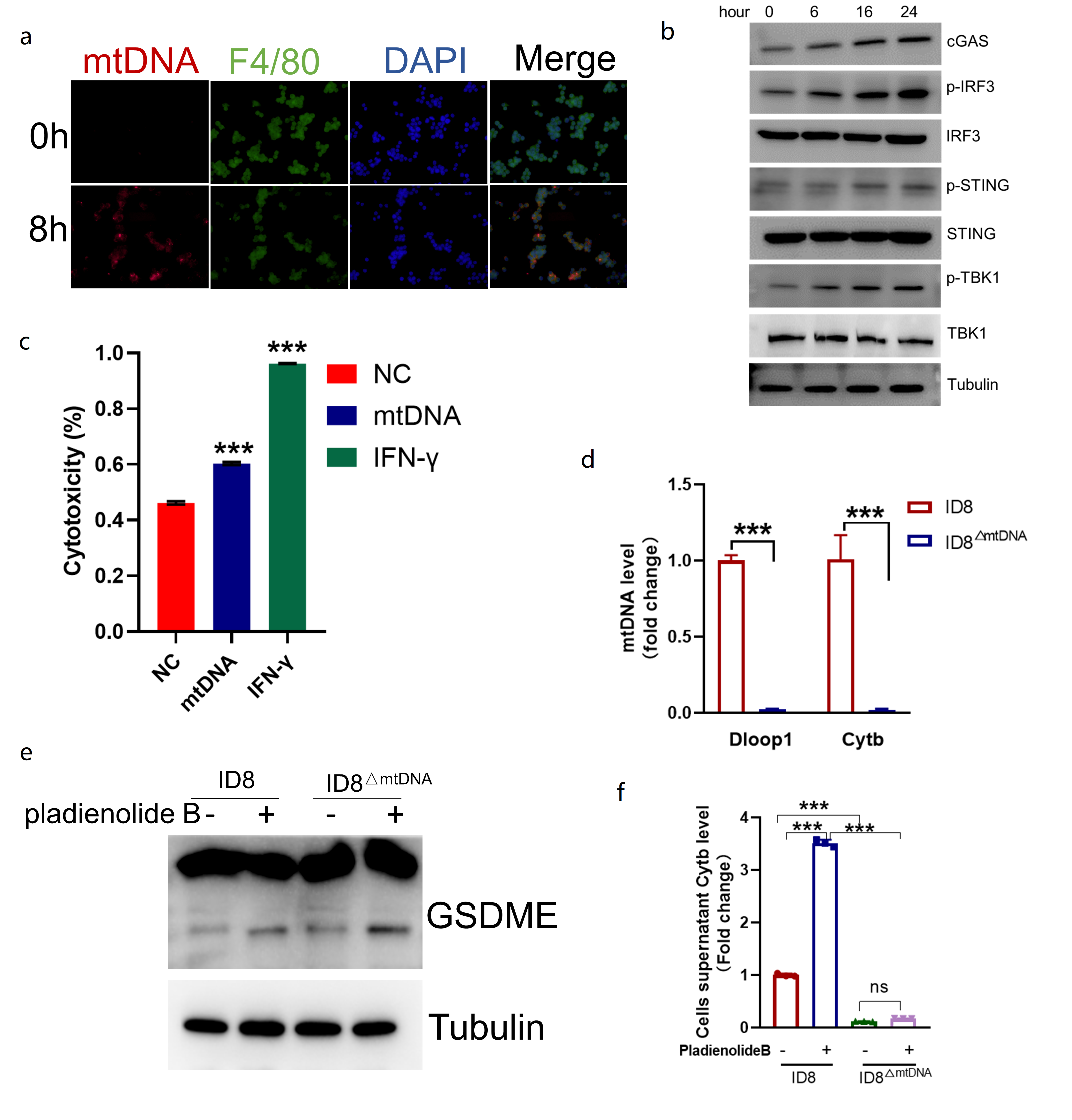


### Figure S6. BMDM engulfs mtDNA and establishment of ID8^△mtDNA^ cells.

1. The phagocytosis of BMDM to cy3-dCTP labelled mtDNA was detected by fluorescence microscopy.
2. cGAS-STING pathway of BMDM at different time of mtDNA treatment was detected by western blot.
3. Cytotoxicity of BMDM treated by mtDNA or IFN-γ were measured by co-culture of BMDM and ID8-LUC with 10:1 effect target ratio. IFN-γ was used as positive control.
4. mtDNA of ID8 and ID8^△mtDNA^ were analyzed by qPCR.
5. GSDME of ID8 and ID8^△mtDNA^ treated with DMSO or pladienolide B was detected by western blot.
6. mtDNA in culture supernatant of ID8 and ID8^△mtDNA^ treated with DMSO or pladienolide B were detected by qPCR. p values were determined by One-Way ANOVA tests. *p < 0.05. **p < 0.01. ***p < 0.001.

(All original blots images could be found in supplementary materials.)
