# Supplementary material for: Inhibition of SF3B1 improves the immune microenvironment through pyroptosis and synergizes with αPDL1 in ovarian cancer
Source: Cell Death Dis. 2023 Nov 27;14(11):775. doi: 10.1038/s41419-023-06301-1 (PMC10682409; doi:10.1038/s41419-023-06301-1)
Supplement: Supplementary file 3 — Supplementary tables [file 41419_2023_6301_MOESM3_ESM.docx]

## Supplementary Tables

### Table S1 Information of antibodies used in the study

| **Antibody** | **Application** | **Company** | **Catalog** | **RRID** | **Dilution** |
| --- | --- | --- | --- | --- | --- |
| SF3B1 | WB | ABclonal | A9737 |  | 1:1000 |
| SF3B1 | RIP， IHC | LifeSpan Biosciences | LS-C179473 |  | RIP:1:100; IHC:1:200 |
| CD8 | IHC | Abcam | Ab237709 | AB_2892677 | 1:1000 |
| P-MLKL | WB | Abcam | ab196436 | AB_2687465 | 1:1000 |
| MLKL | WB | Abcam | ab184718 | AB_2755030 | 1:1000 |
| Tubulin | WB | ABclonal | A12289 | AB_2861647 | 1:1000 |
| PARP | WB | CST | 9542 |  | 1:1000 |
| GSDME | WB | Abcam | ab215191 | AB_2737000 | 1:1000 |
| GSDMD | WB | Abcam | ab215203 | AB_2916166 | 1:1000 |
| NLRP3 | WB | Abcam | ab263899 | AB_2889890 | 1:1000 |
| Csapase3 | WB | CST | 9662 |  | 1:1000 |
| BCL2L2 | WB | ABclonal | A1158 | AB_2758609 | 1:1000 |
| cGAS | WB | ABclonal | A8335 | AB_2770305 | 1:1000 |
| STING | WB | CST | 13647 |  | 1:1000 |
| p-STING | WB | CST | 72971 |  | 1:1000 |
| TBK1 | WB | ABclonal | A2573 | AB_2764459 | 1:1000 |
| p-TBK1 | WB | CST | 5483 |  | 1:1000 |
| IRF3 | WB | ABclonal | A19717 | AB_2862750 | 1:1000 |
| p-IRF3 | WB | CST | 29047 |  | 1:1000 |
| GAPDH | WB | ABclonal | A19056 | AB_2862549 | 1:1000 |
| PD-L1 | WB | Abcam | ab213524 (human),  ab213480 (mouse) | AB_2857903,  AB_2773715 | 1:1000 |
| PD-L1 | IHC | Abcam | ab233482 | AB_2811045 | 1:100 |
| F4/80 | IF | ABclonal | A18637 | AB_2862375 | 1:100 |
| CD3 | FC | BioLegend | 100219 | AB_1732068 | 1:100 |
| CD4 | FC | BioLegend | 100433 | AB_893330 | 1:100 |
| CD25 | FC | BioLegend | 162105 | AB_2894643 | 1:100 |
| CD45 | FC | BioLegend | 103115 | AB_312980 | 1:100 |
| FOXP3 | FC | BioLegend | 126403 | AB_1089118 | 1:100 |
| CD8 | FC | BioLegend | 100705 | AB_312744 | 1:100 |
| IFN-γ | FC | BioLegend | 505809 | AB_315403 | 1:100 |
| CD11b | FC | BioLegend | 101215 | AB_312798 | 1:100 |
| F4/80 | FC | BioLegend | 123107 | AB_893500 | 1:100 |
| CD163 | FC | BioLegend | 156703 | AB_2860724 | 1:100 |
| CD86 | FC | BioLegend | 105027 | AB_893420 | 1:100 |
| Ly-6G | FC | BioLegend | 127613 | AB_1877163 | 1:100 |

### Table S2 Primer sequence of RIP-PCR

| Site1-F | TTCTTTGTCTTTGGGGCTGC |
| --- | --- |
| Site1-R | TTCCCAACATAGCTGCCTCA |
| Site2-F | ACTGTGTGCTGAGAGTGTCA |
| Site2-R | GAGACCAGCTTTGCAGAAGG |

### Table S3 Primer sequence for qPCR

| hSF3B1-F | CGTCTGTGTGTTCGAGTGGA |
| --- | --- |
| hSF3B1-R | GCCCACTCCTTGAGCTTCAT |
| hGAPDH-F | CAGAACATCATCCCTGCCTCTAC |
| hGAPDH-R | TTGAAGTCAGAGGAGACCACCTG |
| BCL2L2-pre-mRNA-F | CTTCTGCAAAGCTGGTCTCC |
| BCL2L2-pre-mRNA-R | ATGAGCCCATCCAGTGTTGA |
| BCL2L2-mRNA-F | GACAAGTGCAGGAGTGGATG |
| BCL2L2-mRNA-R | AAGGCCCCTACAGTTACCAG |
| hCYTB-F | TTCACCAGTCAAAGCGAACT |
| hCYTB-R | ATCGGGATGTCCTGATCCAAC |
| hTERT-F | TCACGGAGACCACGTTTCAAA |
| hTERT-R | TTCAAGTGCTGTCTGATTCCAAT |
| mCytb-F | GCTTTCCACTTCATCTTACCATTTA |
| mCytb-R | TGTTGGGTTGTTTGATCCTG |
| mDloop1-F | AATCTACCATCCTCCGTGAAACC |
| mDloop1-R | TCAGTTTAGCTACCCCCAAGTTTAA |
| mTert-F | CTAGCTCATGTGTCAAGACCCTCTT |
| mTert-R | GCCAGCACGTTTCTCTCGTT |
| mNos2-F | CCCCGCTACTACTCCATCAG |
| mNos2-R | CCACTGACACTTCGCACAAA |
| mCd86-F | CGGATGGTGTGTGGCATATG |
| mCd86-F | CTTAGAGGCTGTGTTGCTGG |
| mCd80-F | TATTGCTGCCTTGCCGTTAC |
| mCd80-F | TATGTGCCCCGGTCTGAAAG |
| mCd163-F | TGTGACCATGCTGAGGATGT |
| mCd163-R | CTCGACCAATGGCACTGATG |
| mTgfb1-F | CCACCTGCAAGACCATCGAC |
| mTgfb1-R | CTGGCGAGCCTTAGTTTGGAC |
| mGapdh-F | AGGTCGGTGTGAACGGATTTG |
| mGapdh-F | GGGGTCGTTGATGGCAACA |
