# Supplementary material for: Inhibition of SF3B1 improves the immune microenvironment through pyroptosis and synergizes with αPDL1 in ovarian cancer
Source: Cell Death Dis. 2023 Nov 27;14(11):775. doi: 10.1038/s41419-023-06301-1 (PMC10682409; doi:10.1038/s41419-023-06301-1)
Supplement: Supplementary file 5 — Original western blots [file 41419_2023_6301_MOESM5_ESM.pptx]

## Slide 1
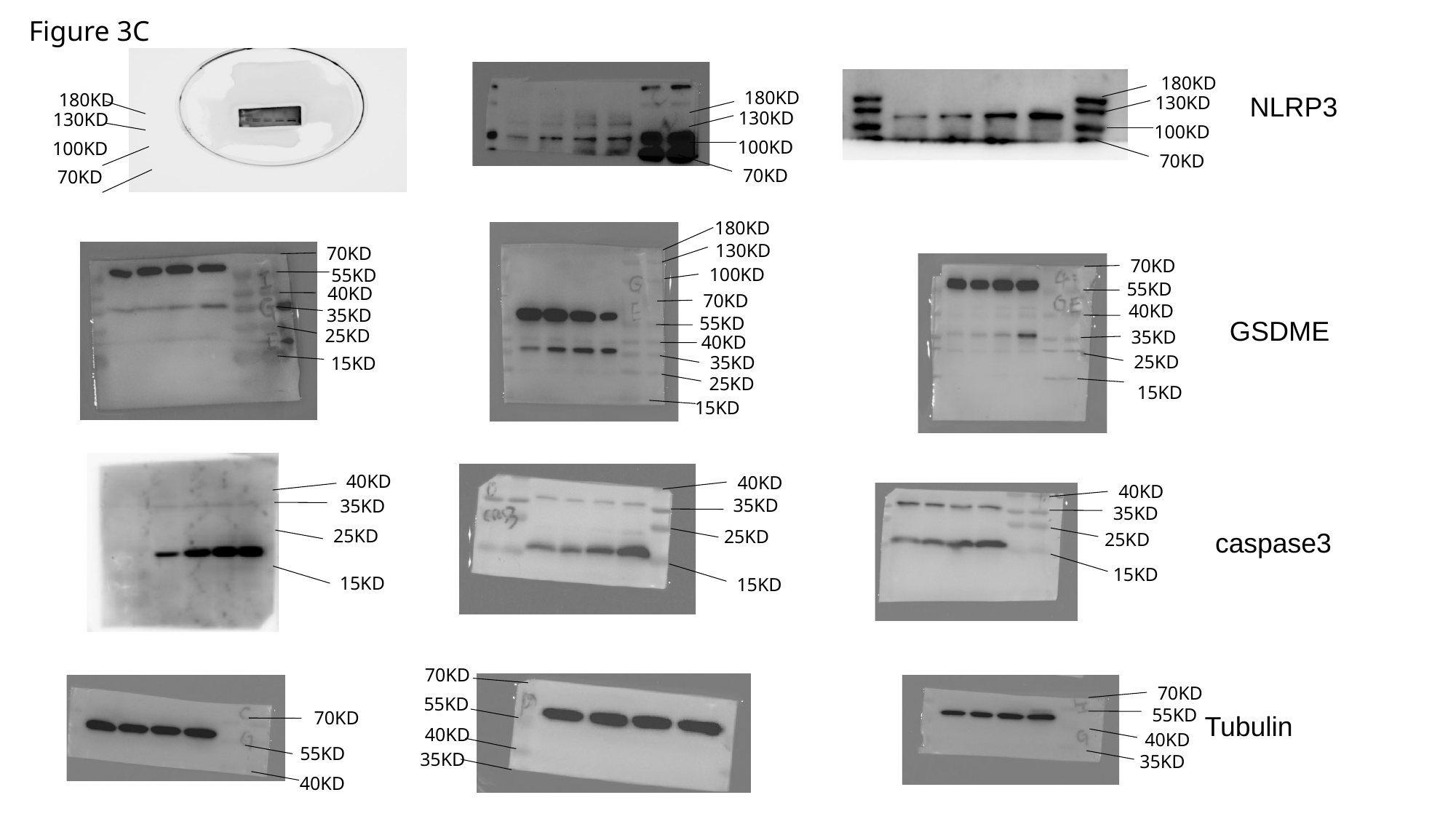

Figure 3C
180KD
130KD
100KD
70KD
180KD
130KD
100KD
70KD
180KD
130KD
100KD
70KD
NLRP3
180KD
130KD
100KD
70KD
55KD
40KD
35KD
25KD
15KD
70KD
55KD
40KD
35KD
25KD
15KD
70KD
55KD
40KD
35KD
25KD
15KD
GSDME
40KD
35KD
25KD
15KD
40KD
35KD
25KD
15KD
40KD
35KD
25KD
15KD
caspase3
70KD
55KD
40KD
35KD
70KD
55KD
40KD
35KD
70KD
55KD
40KD
Tubulin

## Slide 2
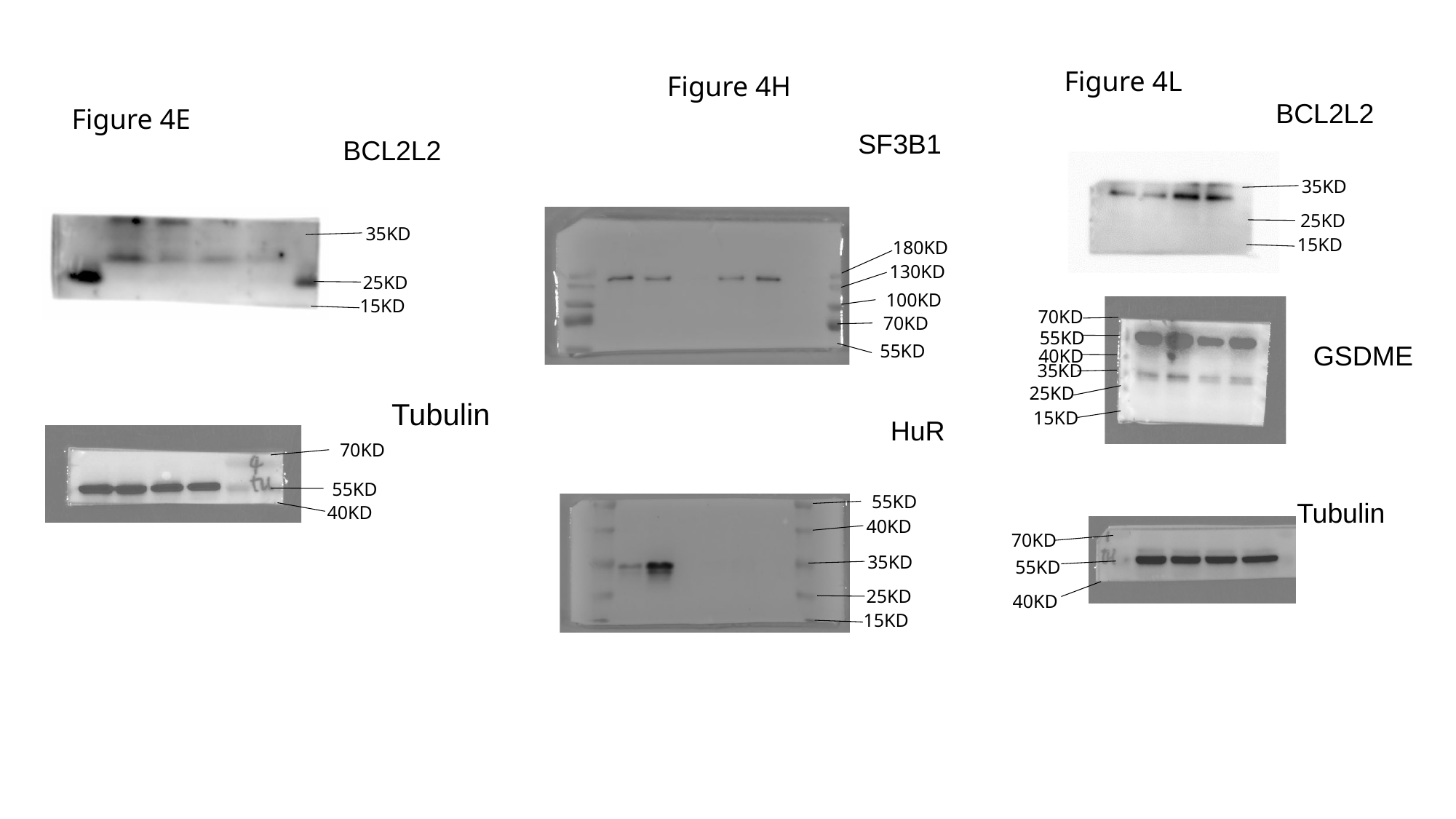

Figure 4L
Figure 4H
BCL2L2
Figure 4E
SF3B1
BCL2L2
35KD
25KD
15KD
35KD
25KD
15KD
180KD
130KD
100KD
70KD
55KD
70KD
55KD
40KD
35KD
25KD
15KD
GSDME
Tubulin
HuR
70KD
55KD
40KD
55KD
40KD
35KD
25KD
15KD
Tubulin
70KD
55KD
40KD

## Slide 3
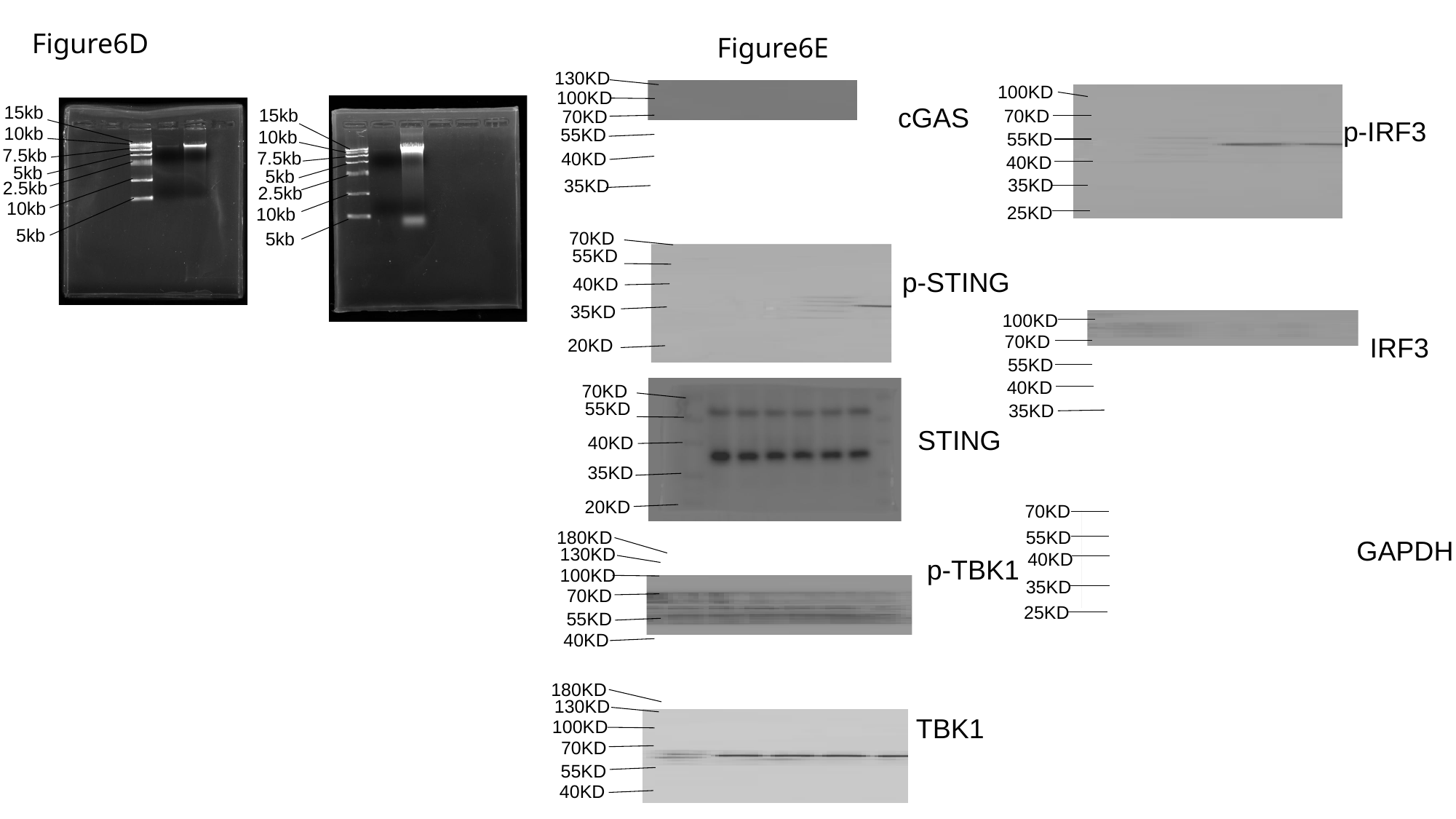

Figure6D
Figure6E
130KD
100KD
70KD
55KD
40KD
35KD
100KD
70KD
55KD
40KD
35KD
25KD
cGAS
15kb
10kb
7.5kb
5kb
2.5kb
10kb
5kb
15kb
10kb
7.5kb
5kb
2.5kb
10kb
5kb
p-IRF3
70KD
55KD
40KD
35KD
20KD
p-STING
100KD
70KD
55KD
40KD
35KD
IRF3
70KD
55KD
40KD
35KD
20KD
STING
70KD
55KD
40KD
35KD
25KD
180KD
130KD
100KD
70KD
55KD
40KD
GAPDH
p-TBK1
180KD
130KD
100KD
70KD
55KD
40KD
TBK1

## Slide 4
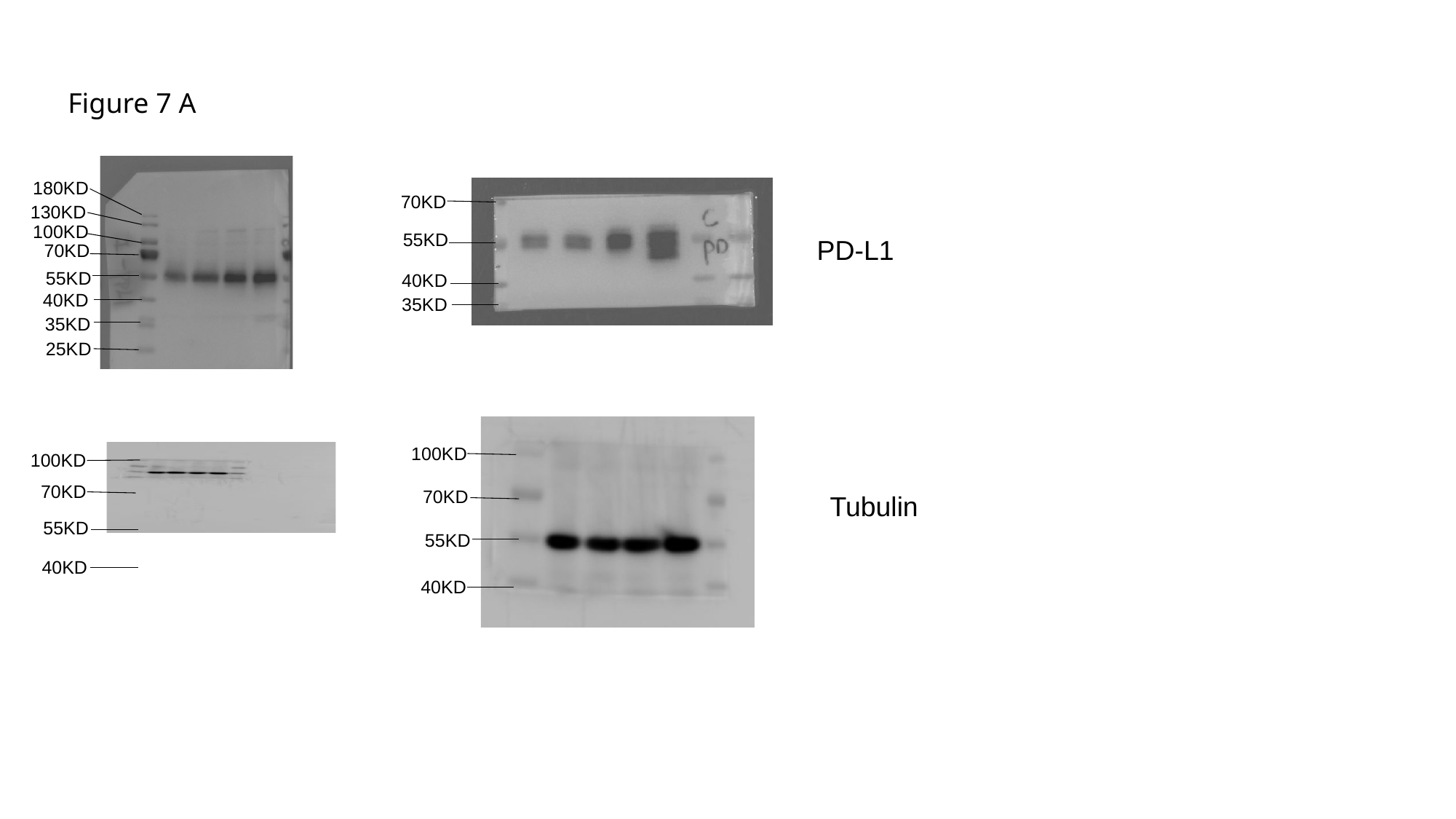

Figure 7 A
180KD
130KD
100KD
70KD
55KD
40KD
35KD
25KD
70KD
55KD
40KD
35KD
PD-L1
100KD
70KD
55KD
40KD
100KD
70KD
55KD
40KD
Tubulin

## Slide 5
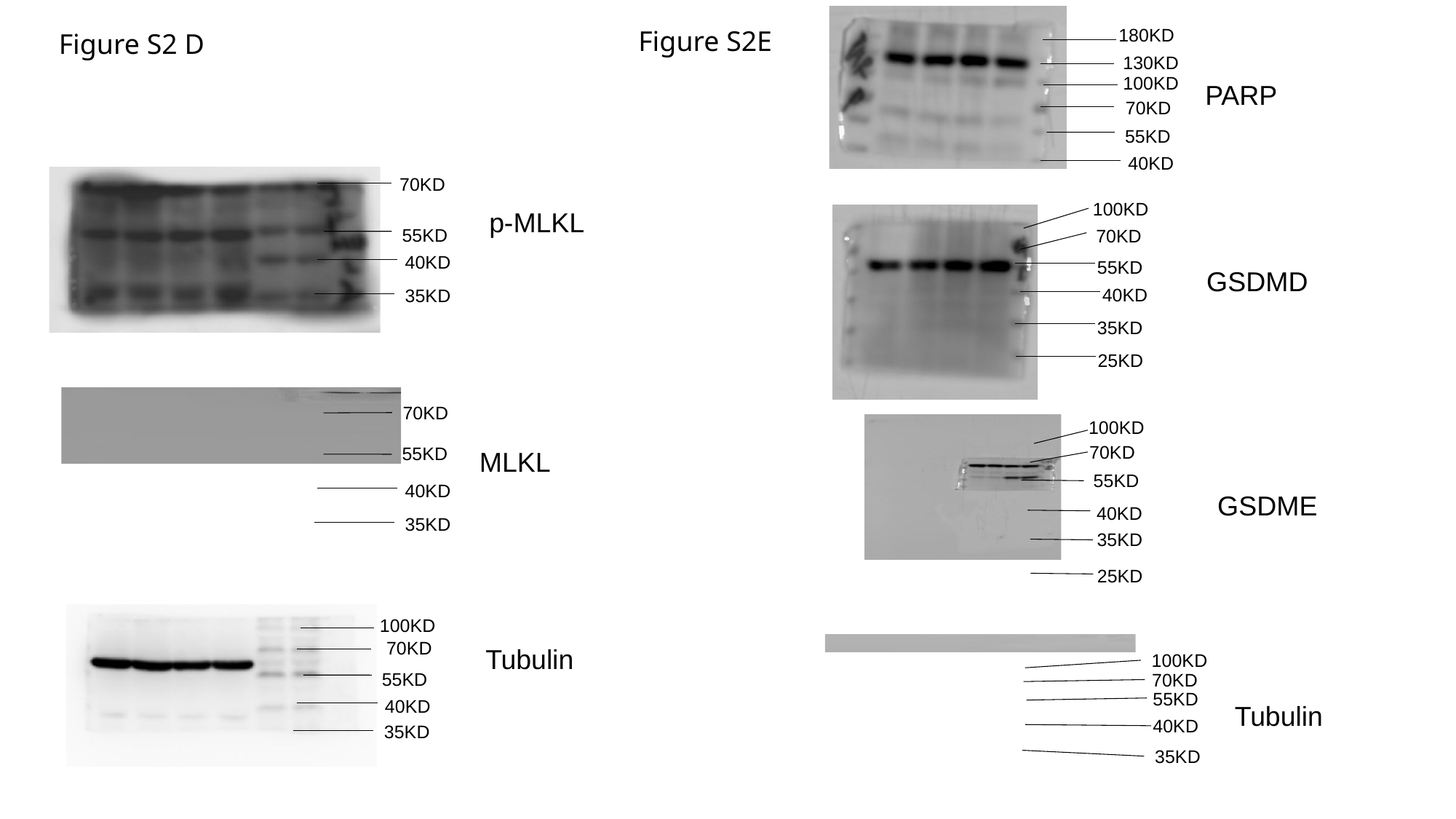

180KD
130KD
100KD
55KD
40KD
70KD
Figure S2E
Figure S2 D
PARP
70KD
55KD
40KD
35KD
100KD
70KD
55KD
40KD
35KD
25KD
p-MLKL
GSDMD
70KD
55KD
40KD
35KD
100KD
70KD
55KD
40KD
35KD
25KD
MLKL
GSDME
100KD
70KD
55KD
40KD
35KD
100KD
70KD
55KD
40KD
35KD
Tubulin
Tubulin

## Slide 6
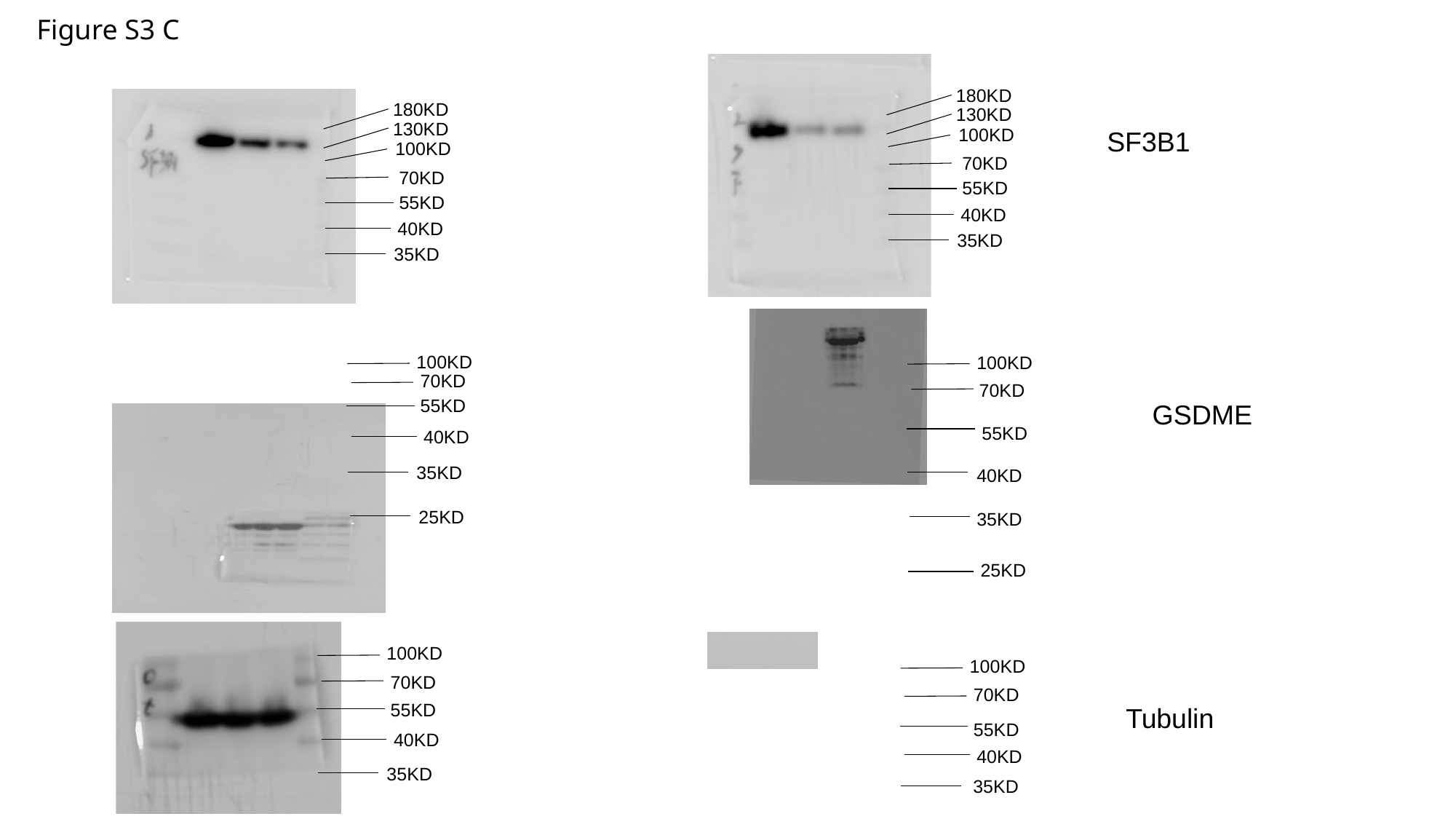

Figure S3 C
180KD
130KD
100KD
70KD
55KD
40KD
35KD
180KD
130KD
100KD
70KD
55KD
40KD
35KD
SF3B1
100KD
70KD
55KD
40KD
35KD
25KD
100KD
70KD
55KD
40KD
35KD
25KD
GSDME
100KD
70KD
55KD
40KD
35KD
100KD
70KD
55KD
40KD
35KD
Tubulin

## Slide 7
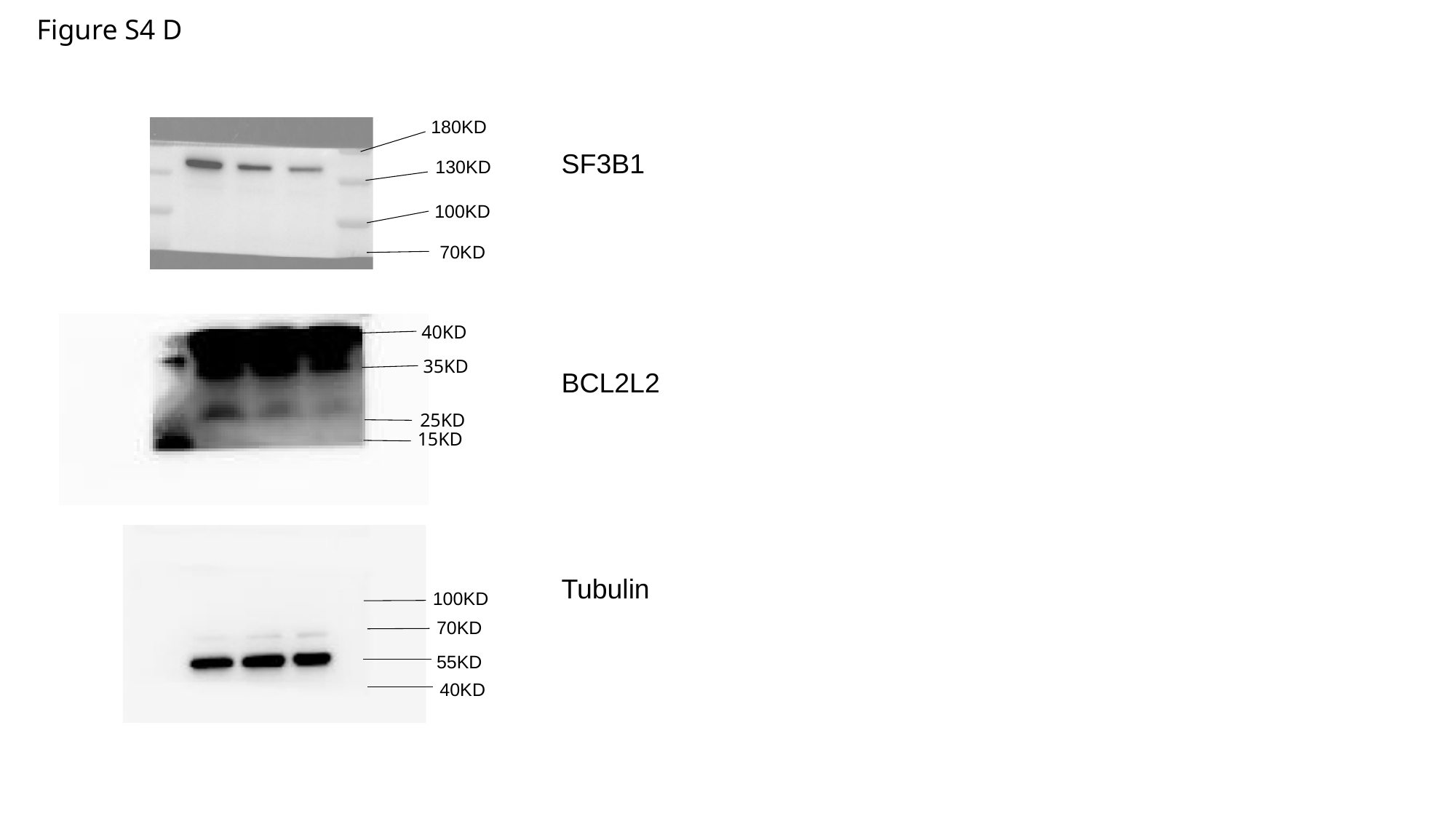

Figure S4 D
180KD
130KD
100KD
70KD
SF3B1
40KD
35KD
25KD
15KD
BCL2L2
100KD
70KD
55KD
40KD
Tubulin

## Slide 8
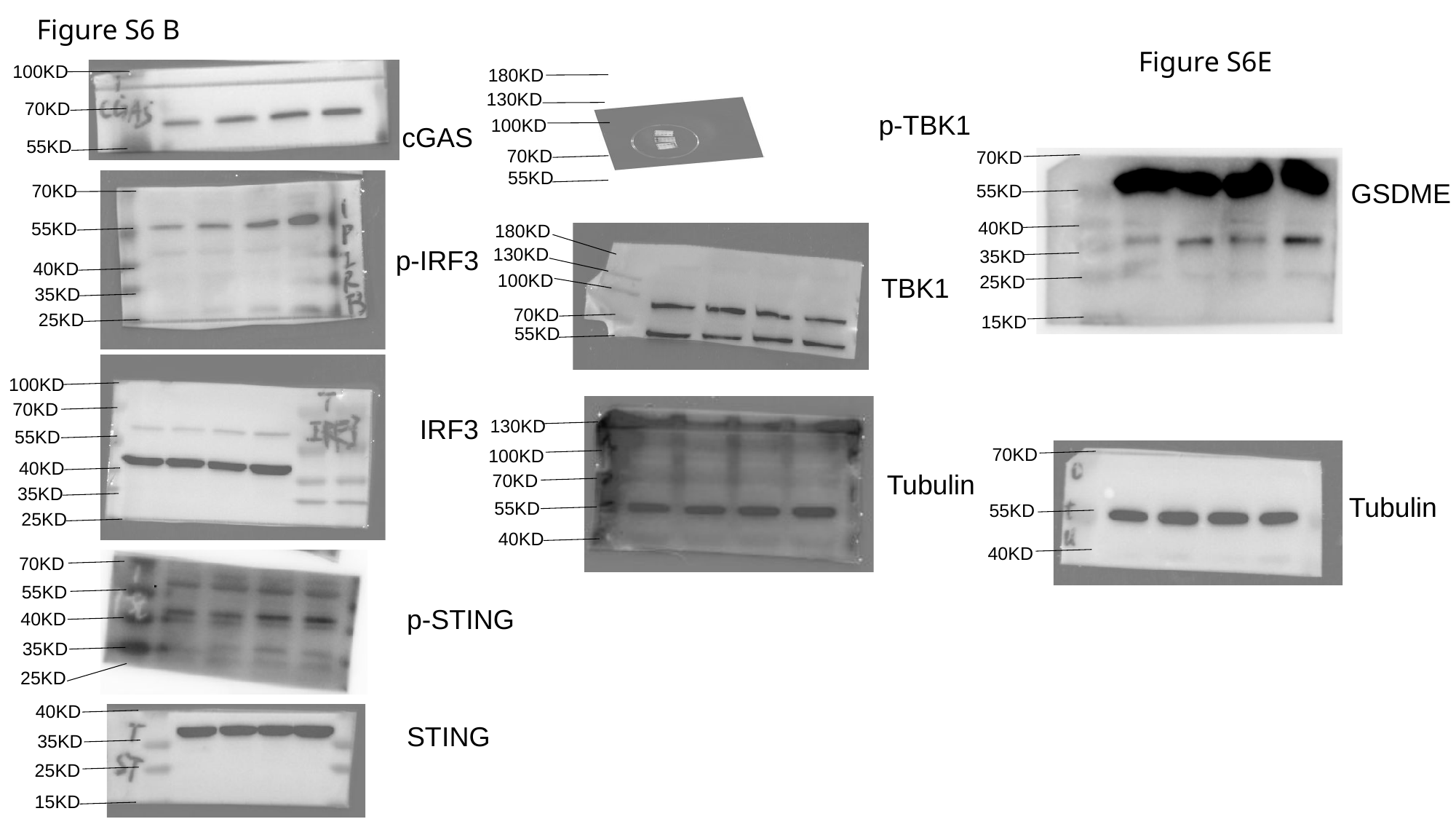

Figure S6 B
Figure S6E
100KD
70KD
55KD
180KD
100KD
70KD
55KD
130KD
p-TBK1
cGAS
70KD
55KD
40KD
35KD
25KD
15KD
70KD
55KD
40KD
35KD
25KD
GSDME
180KD
130KD
100KD
70KD
55KD
p-IRF3
TBK1
100KD
70KD
55KD
40KD
25KD
35KD
130KD
100KD
70KD
55KD
40KD
IRF3
70KD
55KD
40KD
Tubulin
Tubulin
70KD
55KD
40KD
35KD
25KD
p-STING
40KD
35KD
25KD
15KD
STING
